# Vaginal Microbiome Research Consortium for Africa: study protocol of a multicentre prospective clinical study to evaluate temporal vaginal microbial composition associated with maintenance of reproductive health in women in South Africa and Kenya

**DOI:** 10.1136/bmjopen-2024-090938

**Published:** 2025-02-22

**Authors:** Brian Ronald Kullin, Serah Gitome, Anna-Ursula Happel, Tanya Pidwell, Mellissa Lefevre, Anda Madikida, Pauline Wekesa, Karabo Mahlangu, James Ochieng, Lydia Awili, Winnie Agolla, Rhoda Otieno, Amos Mutharimi, Yacoeb Ganief, Rezeen Daniels, Anika Chicken, Kirsten Welp, Hannah Livingstone, Caleb Swanepoel, Shantelle Claassen-Weitz, Pride Kanyoka, Jacques Ravel, Michael Humphrys, Lisa Bilski, Nicola Mulder, Linda-Gail Bekker, Katherine Gill, Heather Jaspan, Elizabeth Anne Bukusi, Jo-Ann Shelley Passmore

**Affiliations:** 1 Department of Pathology, UCT Institute of Infectious Disease and Molecular Medicine, Cape Town, Western Cape, South Africa; 2 Centre for the Aids Programme of Research in South Africa, Durban, KwaZulu-Natal, South Africa; 3 Kenya Medical Research Institute, Nairobi, Nairobi County, Kenya; 4 University of Cape Town Desmond Tutu HIV Centre, Cape Town, Western Cape, South Africa; 5 Department of Microbiology and Immunology, University of Maryland Institute for Genome Sciences, Baltimore, Maryland, USA; 6 Department of Integrative Biomedical Sciences, UCT Institute of Infectious Disease and Molecular Medicine, Cape Town, Western Cape, South Africa; 7 Department of Medicine, UCT Institute of Infectious Disease and Molecular Medicine, Cape Town, Western Cape, South Africa; 8 UCT Institute of Infectious Disease and Molecular Medicine, Cape Town, Western Cape, South Africa; 9 Seattle Children’s Research Institute, Seattle, Washington, USA; 10 Departments of Global Health and Pediatrics, University of Washington, Seattle, Washington, USA; 11 Departments of Global Health and Obstetrics and Gynecology, University of Washington, Seattle, Washington, USA

**Keywords:** Observational Study, IMMUNOLOGY, MICROBIOLOGY, Human Papillomavirus Viruses, HIV & AIDS

## Abstract

**Abstract:**

**Introduction:**

The Vaginal Microbiome Research Consortium for Africa (VMRC4Africa) study is a multicentre observational cohort study. We aim to enrol parallel cohorts of 100 women from two sites in two African countries (N=200) (Desmond Tutu HIV Centre [DTHC], South Africa; Kenya Medical Research Institute [KEMRI], Kenya) to evaluate detailed temporal fluctuations in vaginal microbiota in young, generally healthy women from Southern and Eastern Africa.

**Methods and analysis:**

Cohorts in Kenya and South Africa will be followed up twice a week for 10 weeks to create detailed profiles of vaginal microbial community state types (CSTs; by 16S rRNA gene sequencing) and fungal communities (by internal transcribed spacer (ITS) sequencing) and to identify women with stable *Lactobacillus crispatus*-dominated microbiota, with no evidence of genital inflammation, as assessed by the measurement of inflammatory cytokines.

**Discussion:**

Through the establishment of this African vaginal sample biorepository, the intention will be to cultivate *Lactobacillus* isolates to create a biobank from which to ultimately select geographically diverse *Lactobacillus* strains with health-promoting characteristics that can be co-formulated into live biotherapeutic products (LBPs) to treat bacterial vaginosis (BV) for women in sub-Saharan Africa.

**Ethics and dissemination:**

The VMRC4Africa study has been granted ethical approval by the Human Research Ethics Committees in South Africa (UCT HREC: 611/2022) and Kenya (KEMRI Scientific and Ethics Review Unit: SERU No. 4569). Deidentified microbial community compositional data will be made available on public databases. Results of the study will be published in peer-reviewed journals.

**Strengths and limitations of this study**

- This is an observational cohort study.
- The study aims to assess the vaginal microbial community dynamics and host inflammation in generally healthy women without bacterial vaginosis through intensive vaginal sampling over 10 weeks.
- The study will be conducted in two sub-Saharan African countries.

## Introduction

 Globally and in Africa, an optimal vaginal microbiota is characterised by the dominance of non-*iners Lactobacillus* species and a vaginal pH <4.5.^1^,^3^ Commensal *Lactobacillus* spp. support mucosal barrier function in the female reproductive tract and protect women from infections by (1) lowering the vaginal pH (while being relatively acid tolerant themselves); (2) competitively antagonising pathogens and pathobionts; (3) stimulating host antimicrobial factor production; (4) supporting genital epithelial barrier integrity; and (5) moderating mucosal immune cell function through the induction of tolerising regulatory T cells.^4^ While several vaginal *Lactobacillus* spp have been shown to lower pH and produce active metabolites (like D- or L-lactic acid (LA)), *Lactobacillus crispatus* appears to most consistently prevent adverse outcomes.^5^ Conversely, *Lactobacillus iners*, which only produces L-lactic acid and additionally encodes a cytolysin (inerolysin), is not able to exert protection and is seldom associated with optimal outcomes.^6^ Studies conducted in the USA suggested that African-American women are less likely to be colonised with *L. crispatus* than their Caucasian American counterparts and tended to have more heterogeneous vaginal microbiota and higher pH.^7^ Studies conducted in women from several African countries (including South Africa and Kenya) revealed that while many women are colonised by *L. crispatus*-dominant vaginal communities and have vaginal pHs ≤4.0 around the time of sexual debut,^8^ this is less common in women during their reproductive years, who tend to transition to *L. iners*-dominated or diverse non-*Lactobacillus*-dominant microbiota.^3 9^

Non-*Lactobacillus*-dominated vaginal microbiota that comprise a wide array of strict and facultative anaerobic bacteria, such as those that occur during episodes of bacterial vaginosis (BV), are associated with several adverse reproductive health outcomes, including an increased risk of acquiring sexually transmitted infections (STIs, including HIV), miscarriage, preterm birth, chorioamnionitis and low birthweight infants.^10^,^13^ Unfortunately, young women in sub-Saharan Africa are disproportionately susceptible to both HIV and STIs, and the high rates of BV in the region contribute greatly to this risk. Although the reasons for this are multifactorial, it is thought that genital inflammation associated with BV plays an important role by recruiting CD4+HIV target cells to the vaginal mucosal environment.^14^ We have shown previously that BV and/or STIs significantly increase cervicovaginal inflammatory cytokine concentrations (including macrophage inflammatory protein (MIP)-1β, RANTES (regulated on activation, normal T-cell expressed and secreted) and tumour necrosis factor (TNF)-α) in South African women.^15 16^ We have also shown that women who later acquired HIV had significantly elevated pre-HIV-acquisition genital tract inflammatory markers (including MIP-1α, MIP-1β, interleukin (IL)-8 and interferon-inducing protein (IP)-10) compared with women who did not acquire HIV despite having similar exposure risks.^17^ Detection of either MIP-1α or MIP-1β in cervicovaginal fluid was associated with more than threefold increased risk for subsequent HIV acquisition. In addition, we showed that genital inflammation and BV undermine topical pre-exposure prophylaxis (PrEP) strategies to prevent HIV acquisition in young South African women.^18 19^

Current treatment guidelines for BV recommend the use of oral metronidazole (400 or 500 mg two times per day for 7 days).^20^ Alternatively, a 2 g stat dose of metronidazole or 300 mg clindamycin two times per day for 7 days can be used. However, while BV treatment leads to a decrease in the abundance and presence of BV-associated species, re-colonisation with *Lactobacillus* spp. is often slow or inadequate, and recurrence rates of BV after treatment are high, with >50% of treated women experiencing recurrence by 6 months.^21^ Several studies have shown a modest reduction in bacterial counts of BV-associated microorganisms after antibiotic treatment of BV, inversely associated with increased relative abundances of *Lactobacillus* spp.^22 23^ Resistance to metronidazole may play a role in protecting some BV-associated bacteria during treatment.^24^ Given the relationship between BV, genital tract inflammation and HIV risk, there is an urgent need to rethink BV treatment strategies.

Another significant challenge facing sub-Saharan African populations is the high prevalence of human papillomavirus (HPV) infections in adolescent girls and young women.^25^ HPV is the main cause of cervical cancer, which is the fourth leading cancer in women globally,^26^ and may contribute towards an increased risk of HIV acquisition.^27^ In countries such as South Africa and Kenya, the prevalence of infections with high-risk HPV genotypes is high.^27 28^ Despite both countries having introduced vaccination campaigns in 2014 and 2019, respectively,^29 30^ vaccine uptake rates are thought to be as low as 10–30% in some settings.^31^ Understanding the epidemiology of circulating HPV genotypes, as well as their persistence, is important to evaluate the effectiveness of current vaccine programmes and inform vaccine coverage.

One strategy to restore and maintain vaginal health that has recently gained increasing attention is the use of live biotherapeutic products (LBPs) that include bacterial strains with proven probiotic benefits as adjunctive therapy during BV treatment, with the goal of promoting the (re-)establishment of stable, resilient *Lactobacillus*-dominant vaginal microbiota. Similar microbiome interventions have shown utility in treating gastrointestinal disorders, where broad (eg, faecal microbiome transplants) and targeted (eg, constructed bacterial consortia formulated into LBPs) strategies have resulted in significant clinical benefit in patients with recurrent *Clostridioides difficile* infection and Crohn’s disease, respectively.^32 33^ Based on the benefits evidenced from these approaches, exploring whether LBPs or microbiome interventions are an avenue to restore vaginal health and improve BV treatment outcomes in women is critical. To date, the efficacy of probiotics to treat BV has been mixed,^34^ probably because the majority of the commercial probiotics tested do not contain species commonly found in the lower reproductive tract of women.^35^ Only one LBP, Lactin-V, which contains a single strain of *L. crispatus*, has been tested and shown some success in a phase 2b clinical trial for the prevention of bacterial vaginosis recurrence.^36^ The successful implementation of vaginal LBP treatment strategies requires a better understanding of the factors involved in vaginal microbiota stability in women living in different environments and from different geographies.

Strategic investment in human microbiome research in high-income countries in the past decade yielded large volumes of publicly available sequence data, biased towards industrialised nations that made these investments.^37^ Less progress has been made in microbiome research to understand the relationship between microbial variability and health in low- and middle-income countries.^38^ The context and geographies of these ‘missing microbiomes’ have important implications for disease management in African populations.^39^ Given the high burden of microbiome-based genital conditions, like BV, among southern African women and the associated need to optimise treatment options for women most in need,^12 40^ it is critical to invest in African-led research, thereby addressing local problems with local capacity.^38^

Among the microbiome research that has been published from cohorts across Africa, including 16S rRNA gene sequencing-based surveys of the bacterial composition of the gut, vagina, lung and oral cavity, African scientists have only been first or senior author on a fifth of the manuscripts, with most of the microbiome research from Africa being led by scientists from the USA.^41^ There are several African Centres of Excellence in HIV prevention with the expertise to conduct high-quality microbiome research in Africa, which will help understand determinants of reproductive health for women on the African continent and ultimately lead to the development of novel products to improve BV outcomes. Furthermore, it is unclear whether protective vaginal *Lactobacillus* isolates from women in North America, Europe or other high-income countries would confer a positive advantage to women in Africa and vice versa. There is thus an urgent need to understand the temporal dynamics of vaginal microbiota in African women across menstrual cycles on the African continent. Identifying African women with stable/resilient vaginal microbiomes will ultimately be critical for cultivating and selecting vaginal *Lactobacillus* strains that will promote vaginal health and treat BV in African women.

### Objectives and hypotheses

Our study aimed to intensively investigate vaginal microbiota stability (bacterial and fungal) in generally healthy, STI and BV-free (Nugent score 0–3^42^) women in South Africa and Kenya over a 10-week period, as well as factors associated with microbiota stability. In addition, the relationship between different vaginal community state types (and their transition dynamics) and the host mucosal surface will be investigated.

One reasonable hypothesis is that health-promoting vaginal *Lactobacillus* strains should be isolated from women with a stable vaginal microbiota who are consistently optimally protected (no adverse FGT symptoms; *L. crispatus*-dominated microbiota; no BV, bacterial STIs and vulvovaginal yeast infections). However, it is likely that geography plays a role, and *Lactobacillus* strains that successfully and stably colonise the vaginas of women from different regions in Africa may not perform equally well in different countries. We hypothesise that the best *Lactobacillus* strain combination for a novel BV treatment for women from different regions would likely include isolates with the greatest phenotypic and genotypic breadth and come from a diversity of populations.

This protocol—a pilot African-led initiative—thus enrols Kenyan and South African women using standardised enrolment criteria and sampling methods to create a biorepository of African vaginal samples for the cultivation of *Lactobacillus* isolates and the development of an LBP for vaginal health that will serve women in sub-Saharan Africa. In particular, we propose that (1) characterisation of vaginal microbiota interactions with the host epithelium and obtaining bacterial isolates from different geographies in Africa will contribute to the global effort to understand the functional microbial diversity that occurs in women with stable *Lactobacillus*-dominant versus unstable vaginal microbiota; (2) vaginal *Lactobacillus* strains from different regions of Africa will have better antimicrobial properties against local BV-associated bacteria (such as *Gardnerella* spp and *Prevotella bivia*); (3) addition of a diverse range of *Lactobacillus* isolates from different regions in Africa into the global sequence database will contribute substantially to our understanding of geographical adaptation of vaginal microbiomes and sequences that are publicly available; and finally (4) designing a LBP which includes a diverse but genetically complimentary consortium of *L. crispatus* and potentially other more global *Lactobacillus* isolates will more likely result in stable *L. crispatus* vaginal colonisation in women in Africa and globally.

### Study design

The Vaginal Microbiome Research Consortium for Africa (VMRC4Africa^43^) study is a multicentre observational cohort study to assess the vaginal microbiota compositional dynamics and host inflammation in generally healthy women without BV over 10 weeks.

## Methods: participants and outcomes

### Study setting

Young women (18–40 years) are enrolled at two sites (Desmond Tutu HIV Centre (DTHC)^44^, Masiphumelele Clinical Research Site (CRS) and the Kenya Medical Research Institute, Kargeno Research and Policy Hub, Kisumu CRS).

The DTHC study site in Cape Town, South Africa serves two peri-urban low-income communities with a substantial burden of HIV. It has easy access to a population of >50 000 people. In close proximity, there are several middle-class suburbs with a combined population of >50 000 people. The site has a long-established relationship with community stakeholders, experienced study staff, sexual and reproductive health services, and an active Community Advisory Board (CAB).

The Kargeno Research and Policy Hub^45^ situated in Kisumu town, Kenya serves Kisumu County and several neighbouring counties in the Lake Victoria region. It conducts trans-disciplinary research in applied public health, public policy, epidemiology, maternal health, social and behavioural science with a specific focus on sexual reproductive adolescent and child health.

### Patient and public involvement

Patients and the public were not formally involved in the design, conduct, reporting and dissemination plans of the research. However, informal involvement through interactions with CABs in each of the communities ensures community engagement during the study procedures.

### Eligibility criteria

At screening, written informed consent (online supplemental figure S3A) is obtained from participants before performing any procedures. In addition, we seek consent that vaginal *Lactobacillus* strains isolated through this study can potentially be included in a commercial formulation to treat BV and for the samples to be stored and shipped to the University of Cape Town (UCT) for further analysis (online supplemental figure S3B). We also seek consent for the isolation of any additional species (*eg, Prevotella* spp, *Gardnerella* spp, etc) for further research purposes. The inclusion and exclusion criteria are listed in Table 1.

### Study visits

Women first attend a screening visit, which includes the collection of vaginal samples for eligibility testing and long-term storage. Participants who are eligible for the longitudinal study then attend three main study clinic visits (enrolment, mid-point and exit), during which they provide an extensive set of vaginal samples and complete detailed behavioural questionnaires. In between the main study visits, participants self-collect vaginal swabs (two per week), which are dropped off at the study sites weekly.

### Participant timeline

**Figure 1:**
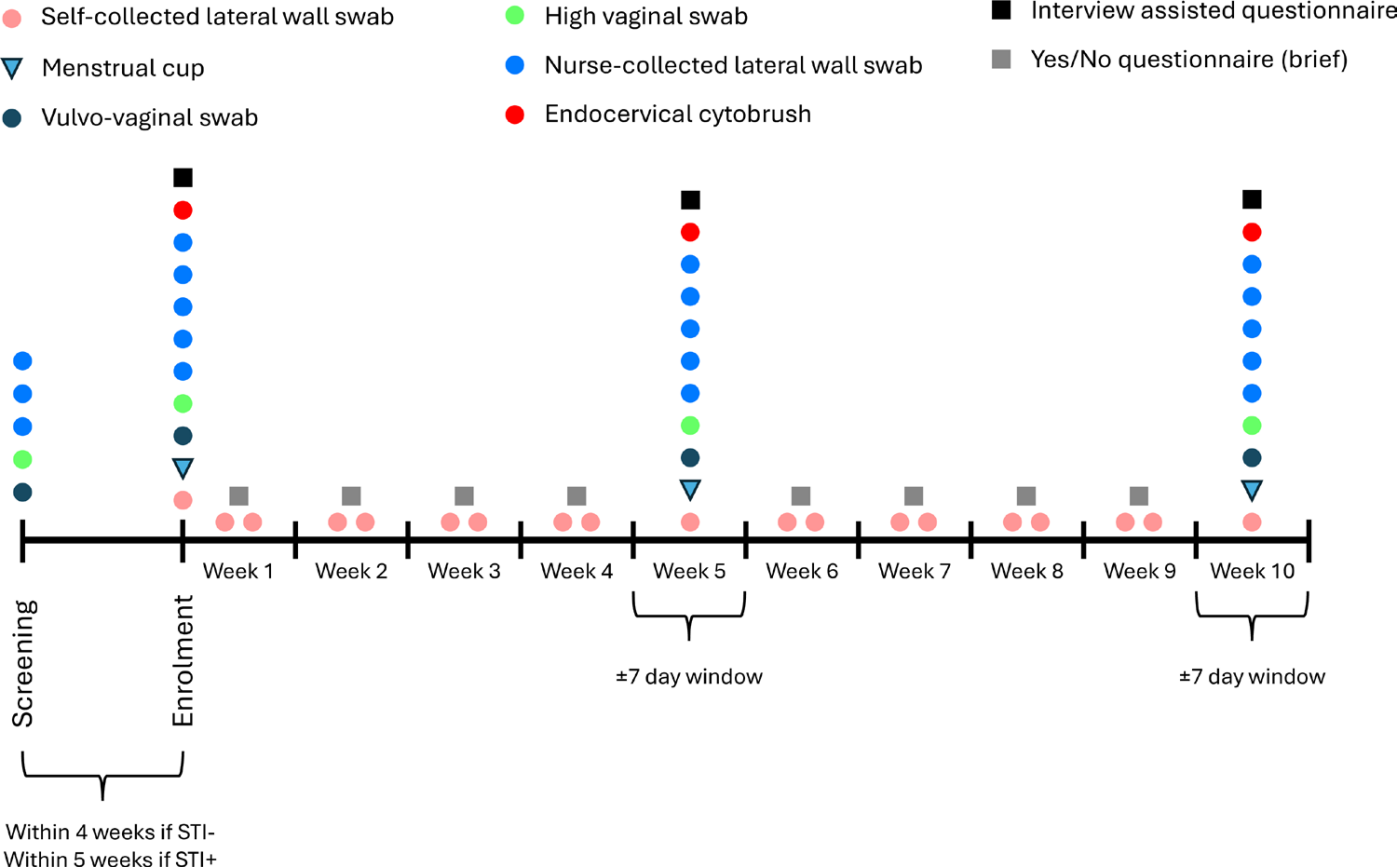
Overview of study design. STI, sexually transmitted infection.

Participant enrolment began in June 2023 and will be completed by the end of February 2025. Participants who meet all the inclusion criteria and none of the exclusion criteria (table 1) are followed up intensively (weekly) for 10 weeks. Eligible participants are enrolled around their menstrual cycle to avoid collection of blood-contaminated genital samples. Those who are deemed eligible at screening but present with recent antibiotic use or treatable STIs will wait 28 days post antibiotic treatment before testing for cure and, if STI-free, enrolment. Thus, screening to exit may be extended to 16 weeks if necessary. The timeline of study visits is shown in figure 1.

**Table 1:** VMRC4Africa inclusion and exclusion criteria

Inclusion criteria	Exclusion criteria
Female at birth	Male at birth
Willing and able to provide informed consent and cognitive ability to understand sampling procedures	BV intermediate or positive by Nugent (Nugent score >3)
Not pregnant	On chronic disease management for gynaecological conditions
HIV negative	Living with an untreated STI*
18–40 years	Currently taking an antibiotic or antibiotic use in the last 4 weeks*
Sexually active for the last 3 months, defined as penetrative penile-vaginal intercourse at least once in the last 3 months	Currently enrolled on any other study prohibiting co-enrolment
Planning to stay in the area for the next 10 weeks	
Willing and able to return for all three nurse visits and return self-collected swabs to the clinic weekly	
Able and willing to provide adequate locator information for study retention purposes	

* If diagnosed with a treatable STI at the screening visit, participants will be treated and can be re-screened for enrolment 28 days after the antibiotic treatment is ended.

STIssexually transmitted infectionsVMRC4AfricaVaginal Microbiome Research Consortium for Africa

### Screening visit

The screening visit starts with a pre-screen (online supplemental figure S1A), which includes an interview with a delegated site staff member as well as rapid tests and pre- and post-counselling for HIV and pregnancy. Participants with symptoms suggestive of gynaecological conditions undergo a targeted physical examination to enable diagnosis and treatment. Non-pregnant participants without HIV and no underlying issues undergo a speculum examination, during which six swab specimens are collected for STI testing, Nugent scoring^42^ and vaginal pH, microbiome, microbial culture and long-term storage (table 2, online supplemental figure S1B). Participants who are eligible for the study based on all screening procedures are invited to an enrolment visit within 4 weeks of the screening examination. This period can be extended to 6 weeks for participants who receive treatment for an STI.

**Table 2:** VMRC4Africa schedule of events

Procedure	The nurse collected				Self-collected
	Screening	Enrolment	Week 5	Week 10	Weeks 1–4, 6–9
Study coordination					
Pre-screen for eligibility	X				
Informed consent	X	X*	X*	X*	
Participant locator info	X	X	X		
Eligibility assessment	X	X			
Questionnaires†					
Detailed, interviewer-assisted	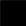	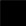	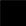	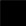	
Yes/no brief questionnaire					1x weekly
Pre-examination tests					
Pre-test counselling	X	X	X	X	
HIV test (blood; rapid)	X	X	X	X	
HIV confirmatory test‡	X§	X§	X§	X§	
Post-test counselling	X	X	X	X	
Pregnancy test (urine; rapid)§	X	X	X	X	
Targeted physical examination	X		X	X	
Sample collection**†					
Lateral vaginal wall swab (self-collected) [SM]		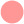	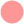	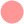	
Speculum examination	X	X	X	X	
Menstrual cup [S]		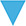	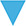	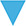	
Vulvovaginal swab [1]§††	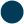	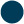	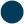	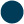	
High vaginal wall swab [2]	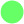	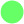	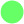	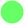	
Lateral vaginal wall swab [3]††	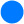	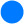	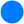	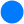	
Lateral vaginal wall swab [4]	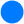	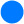	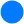	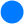	
Lateral vaginal wall swab [5]	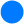	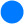	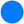	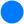	
Lateral vaginal wall swab [6]		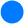	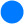	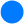	
Lateral vaginal wall swab [7]		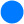	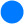	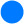	
Endocervical cytobrush [8]		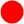	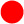	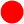	
Lateral vaginal wall swab (self-collected)					2x weekly¶

* Record continuing consent for procedures;.

† Symbol shapes and colours match the collection symbols in figure 1.Swabs not stored for further analyses.

‡ Confirmatory HIV test only for positive HIV rapid test;.

§ Referral for pregnancy, HIV+ and symptomatic STI/BV detection., Participants with STIs at screening will attend a post-treatment visit to confirm test-of-cure prior to enrolment;.

¶ 2x self-collected swabs in the week on different days. To be returned to the clinic for storage weekly and batch shipping twice during the study.

** Numbers in square brackets match ‘Collection label’ in Table 3table 3;.

†† Swabs not stored for further analyses.

BVbacterial vaginosisSTIssexually transmitted infectionsVMRC4AfricaVaginal Microbiome Research Consortium for Africa

**Table 3:** Sample collection types and storage media

Collection order	Specimen	Collection label	Intended purpose	Collection device	Storage media
1	Lateral vaginal wall (self-collected)	SM*	Microbiome	Flocked swab	Qiagen C2.1 buffer
2	SoftDisk†	S‡	Cytokines, mucus assays, HPV	SoftDisk	PBS
3	Vulvo-vaginal	1	STI testing	Flocked swab	Dry/Xpert buffer‡
4	High vaginal wall	2	Storage	Flocked swab	Dry
5	Lateral vaginal wall	3	BV Nugent, pH	Flocked swab	Fixed slide for Nugent scoring
6	Lateral vaginal wall	4	Bacterial+fungal culture	Copan Eswab	Transport media+glycerol
7	Lateral vaginal wall (nurse-collected)	5*	Microbiome	Flocked swab	Qiagen C2.1 buffer
8	Lateral vaginal wall†	6	Proteomics	Flocked swab	Dry
9	Lateral vaginal wall†	7	Metabolomics	Flocked swab	Dry
10	Endocervical cytobrush†	8	Transcriptomics	Qiagen DiGene careBrush	RNALater buffer

* The self-collected microbiome swab is collected by the participant prior to any examination procedures, whereas the nurse-collected microbiome swab is collected during the speculum examination.

† At enrolment, week 5 and exit visits only.

‡ The SoftDisk is used to collect vaginal secretions.

STIssexually transmitted infections

### Enrolment, midpoint and exit visits

The enrolment visit (online supplemental figure S1C) starts with an interview with the clinician as well as rapid tests for pregnancy and HIV. Prior to the speculum examination, participants are asked to self-collect a vaginal swab. This provides an opportunity for the clinician to teach the participant how to self-collect swabs, and the swab will later be used in a comparison between self-collected and nurse-collected swabs for microbiome analysis. A menstrual collection cup (SoftDisk) is then inserted for 30–90 min to collect vaginal secretions, while the participants are asked to complete the more detailed questionnaires. This is followed by a speculum examination with the study nurse, during which the menstrual cup is removed and a total of eight swabs are collected (table 2) for STI testing, Nugent scoring^42^ and vaginal pH, microbiome, culture and long-term storage for future studies. Midpoint and exit visits proceed as for the enrolment visit, with the same samples being collected (online supplemental figures S1C and D).

### Weekly visits

Between the three main study visits, participants self-collect two vaginal swabs weekly on separate days for microbiome analysis. Participants are provided with an instructional pamphlet with each swab set. Participants visit the clinic once a week to drop off the self-collected swabs and pick up the next set of swabs. At this visit, they also complete a short questionnaire noting which day each swab was collected and reporting recent sexual activity, medication taken and menses information. If participants are not able to drop off their samples, members of the project staff can collect them from their homes.

### Outcomes

The anticipated outcomes are as follows:

1. A detailed description of vaginal microbiota compositional dynamics (bacterial and fungal) across approximately two menstrual cycles for 200 enrolled women in South Africa and Kenya.
2. Longitudinal cytokine data for these women that can be used to investigate the association between vaginal microbiota compositional shifts and host inflammation.
3. HPV prevalence and diversity data.
4. Establishment of an Africa-led collaboration between Centres of Excellence in Africa with the necessary skills in conducting reproductive health-focused clinical trials across the continent and ability to generate microbiome data.
5. Creation of a biorepository of genital tract samples and standardised metadata (including extracted DNA from 5000 self-collected vaginal swabs and 4800 cervicovaginal swabs from screened and enrolled women) that can be used for future studies.

### Sample size

Previously, we have shown that approximately 40–45% of women participating in longitudinal studies in the region had a BV Nugent score <4 (BV negative) at enrolment.^3 42 46^ Of these women, 60% remained BV negative across three longitudinal study visits. However, of the persistently BV-free women, only ~30% had a non-*iners Lactobacillus*-dominant vaginal microbiota across all visits. We aim to enrol a total of 200 women in this study, with 100 women at each study site. We estimate that to enrol 200 women, we will need to screen a total of 600 women (n=300 women at each site). Assuming that the proportions reported in our previous studies are preserved over time, we expect that of the 200 enrolled women, ~120 out of 200 will remain persistently BV-free and approximately 40 women (n=20 per site) will have broadly longitudinally stable non-*iners Lactobacillus*-dominant communities.

### Recruitment

Participants are recruited via the CRS standard recruiting processes. Participants are informed about the study via social media, flyers, posters and word of mouth. Each site uses a variety of recruitment approaches that work best for the local setting, but, in general, they include community health promoters as recruiters, community events and mobilisation, partnerships with appropriate programmes and via popular social media platforms. Recruitment materials educate women about HIV, sexual health and risks in their community, the effectiveness of PrEP for HIV prevention and the benefits of HIV prevention services. Recruitment will occur over approximately 15 months.

### Methods: data collection, management and analysis

#### Questionnaires and daily diaries to record exposure to sex and antibiotics

A detailed interviewer-assisted questionnaire is administered assessing demographics, behaviour, sexual and vaginal practices, menstrual and health history, contraceptive choice, based on questionnaires from our previous studies in the same populations.^47^ A standardised menstrual practices questionnaire is completed at enrolment and exit to capture information regarding sanitary care (including sanitary pads, cloths, newspaper, menstrual cups and/or tampons) during menstruation (online supplemental figure S2).^48^ In addition, a daily diary modelling those previously used by Gajer *et al*^49^ in the form of a yes/no check-off list is completed weekly to capture menstrual bleeding, sexual activity, use of medications, contraceptives or diaphragm. Data are captured using RedCap.

### Biological sample collection and processing

#### Pregnancy and HIV testing

A urine pregnancy test is used to identify raised beta-human chorionic gonadotrophin (Homemed hCG Pregnancy Strip Ultra-Sensitive (mIU/mL) test strip). HIV testing is conducted using a single-use rapid immunoassay for the qualitative detection of antibodies to HIV-1 and HIV2, which is intended for use in point-of-care settings (Abbott Determine HIV Early Detect). In cases where the screening test is reactive, a confirmatory test using a different test as per the country’s HIV testing guidelines is used (OnSite HIV Ag/Ab 4th Gen. Rapid Test).

### Additional screening tests

Participants who attend the screening visit, but who are not enrolled in the longitudinal study due to having BV, still provide samples during their screening examination. Once approximately 100 such participants (screened but not enrolled) have been screened at each site, one or more additional pre-screening tests will be included to enrich for participants without BV. Options include Amsel criteria testing, a BV Blue rapid test based on sialidase detection and a vaginal pH test. This will be done during the physical examination after the pregnancy and HIV tests and prior to the speculum examination. Participants who test positive for three or more of the Amsel criteria and/or positive for the BV Blue rapid test and/or have a vaginal pH >4.5 will be screened out and will not undergo the speculum examination.

### Speculum examination

The speculum examination will include a physical examination of the vulva and vagina as well as the cervix. During the examination, the clinician checks for any abnormalities that may be indicative of cancer, sexually transmitted disease or other benign conditions. Samples are not collected when participants are on their menses or when there are visible signs of blood on the cervical os. To avoid sample contamination, only warm water is used as a lubricant when inserting the speculum and during the examination. Swabs are collected in the order outlined in table 2, aiming to collect each swab from a different location within the lateral vaginal wall to collect as much fluid as possible.

### Sample processing and storage

Swabs obtained during the speculum examination are processed and stored at the clinical site within 2 hours of collection. Self-collected swabs are stored on receipt at the clinical site. Storage conditions for each sample type are shown in table 3. Where informed consent for sample storage and future use is obtained, participant swabs will be stored in cryobanks at −80°C for further research at each of the participating institutions. Future planned research includes the isolation and characterisation of *Lactobacillus* spp from participants with stable, optimal vaginal microbiota as well as representative BV-associated bacterial species from participants whose vaginal microbiota transition from an optimal *Lactobacillus*-dominated to a non-optimal state. Swabs will also be collected that allow for future transcriptomic, proteomic and metabolomic studies.

### Laboratory processing and analysis

#### STI testing and Nugent scoring

Vulvovaginal swabs are tested in-country for *Chlamydia trachomatis*, *Neisseria gonorrhoeae*, *Trichomonas vaginalis* (SA Clinical site, GeneXpert) and additionally for *Mycoplasma genitalium* (Kenyan clinical sites, GeneXpert). The *M. genitalium* GeneXpert test is not available in South Africa, and it is not routinely tested for given the low prevalence of the infection in asymptomatic women in the region.^50^ Lateral vaginal wall swabs smeared on microscope slides will be used to screen for BV (by Nugent scoring)^42^ and yeast (hyphae and spores) by Gram strain and the vaginal pH determined by spotting onto pH strips (pH 3.6–6.1).

#### HPV genotyping

SoftDisk pellets will be used for HPV genotyping and to run Hybrispot HPV Direct flow chip assays. Specimens will be shipped to the University of Cape Town for HPV genotyping. DNA will be extracted using the MagNA Pure Compact Nucleic Acid Isolation Kit I (Roche, USA) and tested for the presence of HPV DNA using the HPV Direct Flow Kit (Master Diagnostica, Spain). The kit detects 36 HPV genotypes, including 18 hr-HPV genotypes: 16, 18, 26, 31, 33, 35, 39, 45, 51, 52, 53, 56, 58, 59, 66, 68, 73 and 82; and 18 lr-HPV genotypes: 6, 11, 40, 42, 43, 44, 54, 55, 61, 62, 67, 69, 70, 71, 72, 81, 84 and 89. To ensure comparability to other HPV assays and labs, the WHO Global HPV DNA proficiency panel provided by the HPV International Reference Laboratory (Sweden) will be run prior to study start.

### Characterisation of vaginal microbiota composition

#### Sample preparation and Illumina 16S iTag library preparation

DNA will be extracted using the PowerMicrobiome DNA kit (Qiagen).^51^ An extraction control (ZymBiomics Microbial Standard #D6300) will be used to validate the extraction process. The 16S rRNA gene V3–V4 region will first be amplified using universal 319F/806R primers,^52^ and quality checked with Bioanalyzer (Agilent). The amplicon will be barcoded in a second PCR using Illumina Unique Dual Indices to eliminate the possibility of barcode hopping. Pooled triplicate samples will be purified with Agencourt AMPure XP beads (Beckman Coulter). Amplicons will be pooled in equal quantities. Purified libraries consisting of ~120 pooled samples will be sequenced with the Illumina MiSeq platform (300 bp paired-end with v3 chemistry). A microbial community DNA standard (ZymoBiomics Microbial Community Standard #D6305) will be used as a positive control, and no template extraction (kit only) and PCR controls will be included as negative controls.

The ribosomal ITS2 region DNA will be amplified using a pool of primers, including ITS3, ITS86, fITS9, ITS9-FW, fITS7, 5.8S-Fun, ITS4 and ITS4-Fun.^53^,^58^ PCR products will be purified with Agencourt AMPure XP beads (Beckman Coulter). An additional round of PCR will be run to add unique dual indices, and the amplicon pool will be purified and sequenced on an Illumina MiSeq instrument (300 bp paired-end reads). A positive extraction control will be used (*Candida*), and a positive amplification control of a commercial standard (ATCC MSA-1010) with added *Candida* species commonly found in the female genital tract will be used.

#### 16S rRNA gene sequence data analysis

The Divisive Amplicon Denoising Algorithm (DADA) 2 algorithm^59^ will be used to infer amplicon sequence variants (ASVs). The SILVA rRNA gene sequence database and the speciateIT classifier will be used for taxonomic assignment.^60^ The resulting phylogenetic tree, ASV table and taxonomic table (16S rRNA sequence counts) combined with relevant metadata will be consolidated into a phyloseq object using the phyloseq R package^61^ to perform community compositional analyses and ordination.

#### ITS sequence data analysis

The RDP Initial Process tool will be used to perform data quality control of raw sequences and to infer parameters for downstream processing. CD-HIT will be used to infer ASVs. The RDP Naïve Classifier trained on the Warcup database, a manually curated subset of sequences from the UNITE database, will be used for taxonomic assignment. Taxa will be reported as both absolute counts and relative abundance (% of total sequences).

#### Rarefaction analysis, diversity estimates and sample ordinations

To assess the quality of microbial diversity sampling, multiple rarefactions at different sequencing depths will be performed. α-Diversity estimates, richness (Chao1), evenness (Simpson’s E) and phylogenetic diversity (Faith’s PD) will be calculated using the R vegan library.^62^ β-Diversity between samples will be estimated by UniFrac distances. Taxonomic profiles of vaginal microbiota will be assigned to CST using VALENCIA, a tool that classifies a sample to a CST based on the sample’s similarity scores to a reference community derived from bacterial taxonomic profiles from 13 161 vaginal samples, including women from different ethnicity, geography and ages.^63^

### Nurse versus self-collected sample analysis

During the main study visits, two swabs are collected for microbiome analysis, one by the participant and one by the nurse during the speculum examination. These will be compared to evaluate the potential differences in bacterial (and fungal) composition between the nurse-collected and self-collected swabs.

### Total 16S rRNA gene copy number measurement

The total number of 16S rRNA gene copies in each DNA sample will be measured using the TaqMan BactQuant assay targeting the V3–V4 region of the gene.^64^ The total number of 16S rRNA gene copies will be expressed as copy per swab and is used as an estimate of bacterial absolute abundance (total count of bacterial cells present in a sample). An estimate of absolute abundance for each taxon in a sample will be calculated by multiplying the total 16S rRNA gene copies obtained by the BactQuant assay and the relative abundance of each taxon identified by 16S rRNA gene sequencing.

### Measurement of cervicovaginal cytokines by Luminex

We will measure inflammatory cytokines in menstrual cup secretions in all women at three visits (enrolment, 5 and 10 weeks). Samples will be stored at −80°C until the assay is performed. Cytokine concentrations in genital secretions will be assessed using Luminex. Data will be collected using a Bio-Plex Suspension Array Reader (Bio-Rad Laboratories) and a 5 PL regression formula used to calculate cytokine concentrations from the standard curves and analysed using BIO-plex manager software (V.4; Bio-Rad Laboratories). As we have previously defined,^17^ genital tract inflammation in this study will be considered as women having >5/9 of the following cytokines/chemokines in the top 75th percentile of the population: IL-1α, IL-1β, IL-6, TNF-α, IL-8, IFN-γ, IP-10, MCP-1, MIP-1α, MIP-1β. All women below the 75th percentile of these cytokines will be considered to be not inflamed.

### Statistical methods

Baseline characteristics, such as demographic and analytical data, will be summarised using descriptive statistical methods. Continuous data will be summarised using the mean, the median, SD and the range (minimum and maximum value). Categorical values will be summarised using frequency counts and percentages. Sensitivity and specificity of tests predicting CST I (*L. crispatus-*dominated vaginal microbiota) will be determined, and Receiver Operator Characteristic (ROC) curves calculated. Positive and negative predictive values will be calculated.

The twice-weekly dynamics of relative abundance of the 30 most abundant taxa and CST distribution will be visualised using stream graph representation, and the measure of microbiota composition constancy over time, as previously performed by Gajer *et al*,^49^ will be applied. The average population-wide deviation from constancy of vaginal microbiota will be calculated for each woman across both sites. In addition, cytokine data will be used to assign genital inflammation categories to women with cytokines in the >75 th percentile of the cohort.^17^ Integration of microbiota and cytokine data will be performed via Bayesian network learning. We will also control for epidemiological variables such as age, site, hormone contraceptive use, STI/BV status during follow-up.

### Data handling

Accurate source documentation and case report forms (CRFs) will be maintained. For each participant screened, an electronic CRF (eCRF) is completed on the RedCap database, even if the participant drops out at any time during the study. For participants who are lost to follow-up, the visits up to the last visit are completed. Reasons for exiting the study prior to completion are recorded. Data entered into the eCRF is, on saving the eCRF, stored in a web-based database, hosted on a secure server at UCT (RedCap). The database has user access control, and all changes are tracked in an audit trail. Further, data quality is ensured by a series of pre-programmed edit checks to ensure plausibility and by manual curation by a professional data manager. The server is subject to regular backups and access control.

### Record retention

The investigator will keep essential study documents (including CRFs/eCRFs) other than participant medical files for at least 15 years after completion or discontinuation of the study. All participant records, laboratory specimens, etc, will be referred to using a scheme that guarantees participant confidentiality and will be kept in a secure storage area with limited access. Each participant will be assigned a pseudonymous identification number. No data that could identify the participant other than this identification number will appear on the CRFs. Participant age is expressed as a range rather than a specific age. Participant sampling series will be expressed as intervals since donation with no specific sampling times reported. Data of study participants will only be used as defined in the informed consent form (ICF) and in line with applicable data privacy regulations. Accordingly, participant records may be reviewed by inspectors of regulatory authorities or ethics committees, who ensure the quality of the study.

An individual’s study data will not be released without the written agreement of the participant, except as necessary for monitoring and auditing by regulatory authorities or ethics committees, or in case of medical emergencies when written consent cannot be obtained, as deemed in the participant’s best interest by the investigator. Results of any tests will not be disclosed to anybody not involved with the study nor to immediate relatives without prior consent of the participant.

### Data availability

#### Type of data

This study includes the collection of data from 16S rRNA gene and ITS sequencing of the vaginal microbiome and mycobiome. Future planned analyses that are beyond the scope of the current protocol include generation of shotgun metagenomic data and whole genome sequence data from pure bacterial isolates. The consent forms approved by the UCT HREC and the KEMRI Scientific and Ethics Review Unit provide for consent for these data to be used for future research purposes and to be shared broadly through unrestricted-access databases in an anonymous fashion without any identifying information.

#### Data repository

The raw sequence data will be deposited in the Sequence Read Archive (SRA) at NCBI, along with all the associated experimental and procedural metadata. The latter outline the manner of sample and library preparation, data collection details, quality scoring metrics for the sequence reads, and processing parameters used to remove barcode and primer sequence. Results will be accessible and open immediately.

## Ethics and dissemination

### Research ethics approval

The study has been approved by research ethics boards at the University of Cape Town (HREC: 611/2022), Kenya Medical Research Institute KEMRI/SERU/4569.

### Participant informed consent

Participants are given an ICF (online supplemental figure 3A) about the study and have the anticipated benefits and the potential risks associated with the protocol procedures explained to them by a delegated staff member. The language used is as non-technical as possible, and the participant is not unduly influenced to participate in the study. Participants are informed that they may voluntarily withdraw from the study for any reason at any time, and a withdrawal will have no negative effects on them receiving standard care afterwards.

An impartial witness is required for the entire informed consent process for any participant who is illiterate or whose literacy is limited. Documentation of the presence of a witness is achieved through their signature on the informed consent document. Illiterate participants indicate their consent via use of their mark (finger/thumb print) on the informed consent documents.

Participants are also asked to provide separate informed consent (online supplemental figure 3B) for the long-term storage of samples after study procedures have been completed. Samples from participants who refuse this will be destroyed after the study ends.

### Incentives and expenses

Participants do not receive any incentives for their participation in the study. However, participants are reimbursed for their time and transport costs to the research clinics. The exact amount is decided by each of the sites and informed by the community advisory committees.

### Additional ethical considerations

Should a participant acquire HIV or become pregnant after enrolment, they are allowed to continue to attend clinic visits and participate in the interviews/questionnaires if they choose to do so. This is to reduce the potential impact of stigma associated with a positive HIV diagnosis and/or unplanned pregnancy. However, no further mucosal samples are collected, since for participants with HIV, this poses an additional risk to study personnel who will need to handle and process potentially infectious samples, and for pregnant participants, the additional discomfort and risk associated with speculum examination and sampling are difficult to justify given that they will not be included in the study analysis.

Participants who become infected with an STI after enrolment are treated on site or referred for treatment but can continue to provide samples should they choose to do so.

The KEMRI and DTHC research groups both maintain active community advisory groups (CAGs) to facilitate community engagement. For KEMRI, the CAG consists of 12 individuals from the same community as the study participants, whereas the DTHC has adult (25–65 years) and youth (15–23) CAGs that consist of 16 individuals each. Both CAGs serve to provide insights into the contraceptive needs of women within the respective communities and how the VMRC4Africa study can address them effectively, while taking into consideration community norms and values. As such, the CAGs are all inclusive outfits with diverse stakeholder and community representation. CAGs serve as advisory and scrutiny bodies, ensuring community participation and feedback in the study, promoting transparency and guiding the study teams on governance and operational matters. Both CAGs meet with the study teams two to three times per year.

### Dissemination of study results

The dissemination strategy and activities will follow the principles and best practices successfully tested by the partners in other projects in both countries. All research results/reports will be duly reviewed, and a copy will be sent to relevant collaborators and partners involved in the project before these are disseminated and/or published. When appropriate, the reports will refer to other research projects and build on the existing results and literature. Research will be conducted following sound analysis and scientific practice principles, considering as much as possible the policy requirements and needs. All public results will be accessible from the project website (https://vmrc4africa.org/home/) and usable for all parties who may benefit from them.

Dissemination activities are planned in accordance with the stages of the project. Several dissemination activities will take place during the first 18 months of the project; the most significant dissemination activities will take place as final research results are available. The dissemination activities are to be performed according to the following logical schedule:

1. Initial awareness phase (prior to enrolment): This establishes community awareness of the VMRC4Africa initiative (posters, pamphlets, peer educators, local press releases and community engagement in a variety of forms) and analysis of relevant information resources in terms of identification of dissemination opportunities.
2. Targeted dissemination phase (post-enrolment): The consortium will enrich their websites, update the project communication kit, attend selected events and organise workshops. Host a participant dissemination event to present the study results.
3. Post-study (month 60–63): This represents the period when VMRC4Africa consortium partners will start dissemination to the scientific community. This phase will also focus on informing the target audience of the VMRC4Africa exploitable outputs.

Publications will be made available through open access that enables the unrestricted access and reuse of all peer-reviewed published research. In line with funder policies, open access publication will be made available through a Creative Commons Attribution 4.0 Generic Licence; publications will be accessible and open immediately (no embargo).

## Discussion

This study aims to pilot the formation of an African network, collecting key information about geographical variation in vaginal microbiota across two African countries. This observational study will serve as a framework that could be adopted by other study teams in other countries from Central and West Africa to form the VMRC4Africa network and broaden our knowledge of the vaginal microbiota across the African continent. Altogether, the study will inform African scientists on the composition, structure and dynamics of vaginal microbiotas and mycobiota, infection with HPV and cervicovaginal inflammation in South African and Kenyan women. In addition, we will build a well-curated biorepository, including samples from women with stable *Lactobacillus*-dominated microbiota from which *L. crispatus* strains with health-promoting properties can be isolated, alongside extensive metadata to support the value of these samples. Ultimately, the VMRC4Africa network will improve our understanding of the impact of geography on the vaginal microbiome and will contribute *Lactobacillus* strains for inclusion in a combination LBP consortium to treat and/or prevent BV, its associated risks, including preterm birth on this continent.

## supplementary material

10.1136/bmjopen-2024-090938 online supplemental file 1
