# Supplementary material for: Vaginal Microbiome Research Consortium for Africa: study protocol of a multicentre prospective clinical study to evaluate temporal vaginal microbial composition associated with maintenance of reproductive health in women in South Africa and Kenya
Source: BMJ Open. 2025 Feb 22;15(2):e090938. doi: 10.1136/bmjopen-2024-090938 (PMC11848679; doi:10.1136/bmjopen-2024-090938)
Supplement: online supplemental file 1 [file bmjopen-15-2-s001.pdf]

Figure S1A

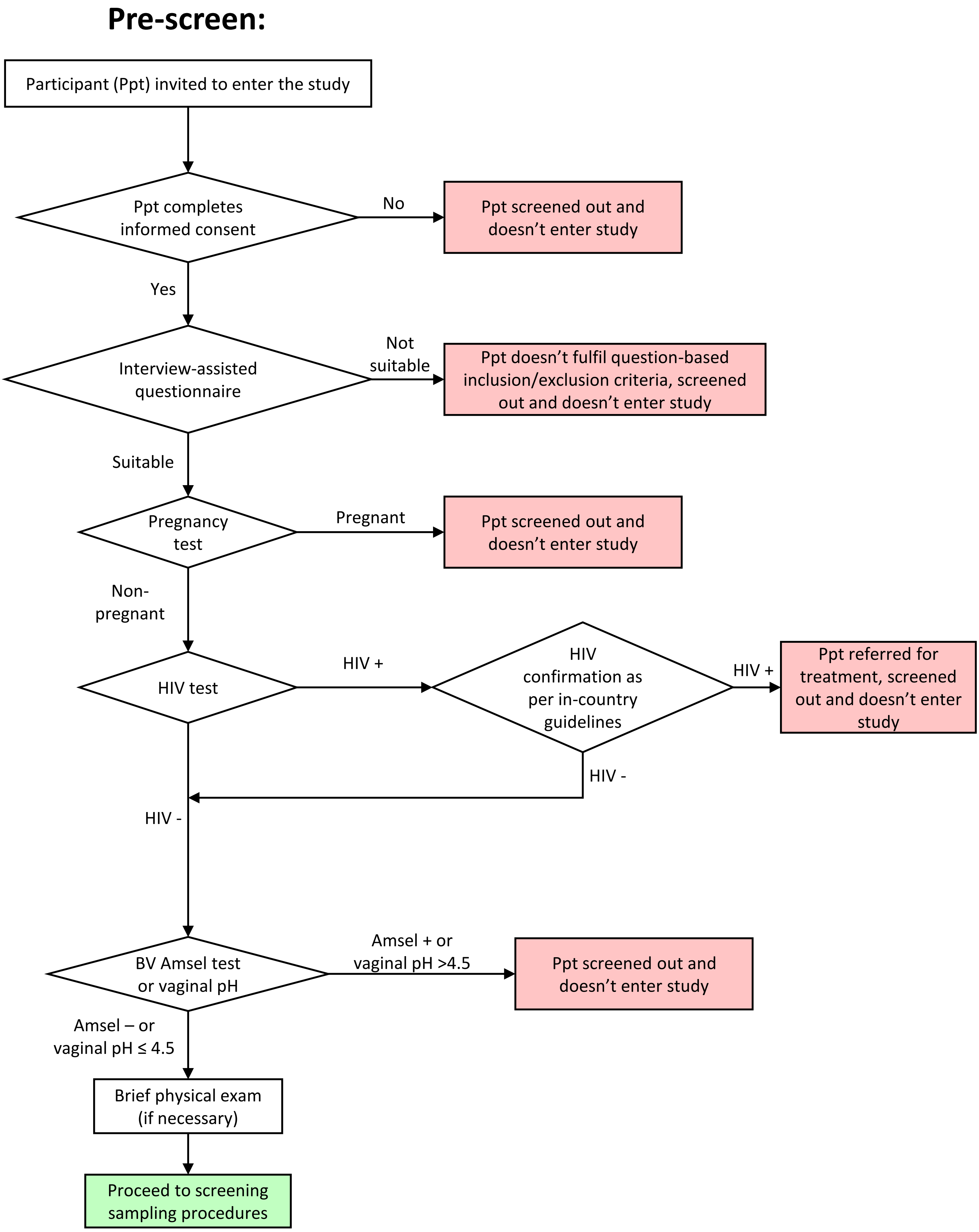
Pre-screen:
Participant (Ppt) invited to enter the study
Ppt completes informed consent
No
Ppt screened out and doesn't enter study
Yes
Interview-assisted questionnaire
Not suitable
Ppt doesn't fulfil question-based inclusion/exclusion criteria, screened out and doesn't enter study
Suitable
Pregnancy test
Pregnant
Ppt screened out and doesn't enter study
Non-pregnant
HIV test
HIV +
HIV confirmation as per in-country guidelines
HIV +
Ppt referred for treatment, screened out and doesn't enter study
HIV -
HIV -
BV Amsel test or vaginal pH
Amsel + or vaginal pH >4.5
Ppt screened out and doesn't enter study
Amsel – or vaginal pH ≤ 4.5
Brief physical exam (if necessary)
Proceed to screening sampling procedures

Figure S1B

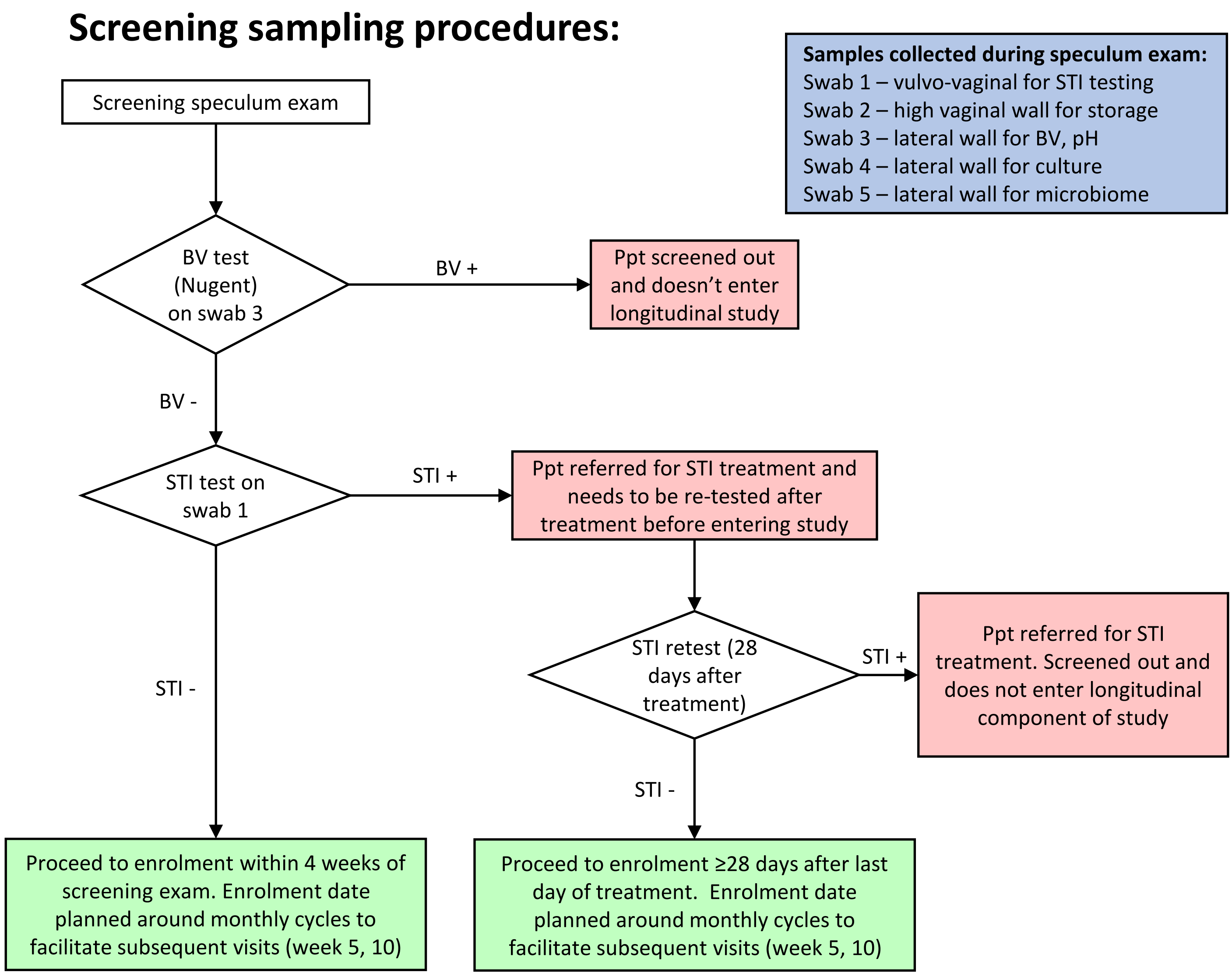
Screening sampling procedures:
Samples collected during speculum exam:
Swab 1 – vulvo-vaginal for STI testing
Swab 2 – high vaginal wall for storage
Swab 3 – lateral wall for BV, pH
Swab 4 – lateral wall for culture
Swab 5 – lateral wall for microbiome
Screening speculum exam
BV test (Nugent) on swab 3
BV +
Ppt screened out and doesn't enter longitudinal study
BV -
STI test on swab 1
STI +
Ppt referred for STI treatment and needs to be re-tested after treatment before entering study
STI -
STI retest (28 days after treatment)
STI +
Ppt referred for STI treatment. Screened out and does not enter longitudinal component of study
STI -
Proceed to enrolment within 4 weeks of screening exam. Enrolment date planned around monthly cycles to facilitate subsequent visits (week 5, 10)
Proceed to enrolment ≥28 days after last day of treatment. Enrolment date planned around monthly cycles to facilitate subsequent visits (week 5, 10)

Figure S1C

Enrolment, mid-point (pre-exam):

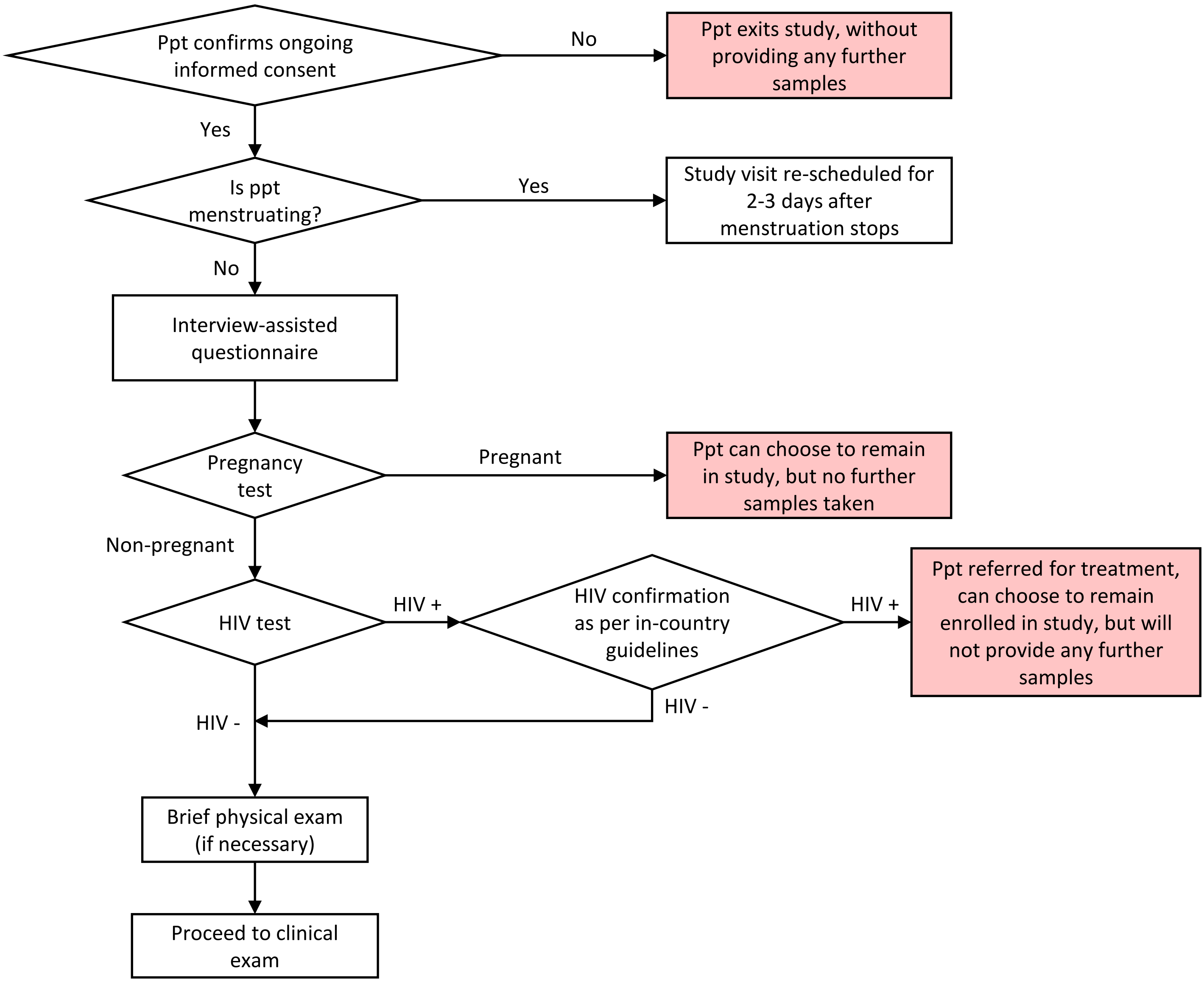

Clinical sampling procedures:

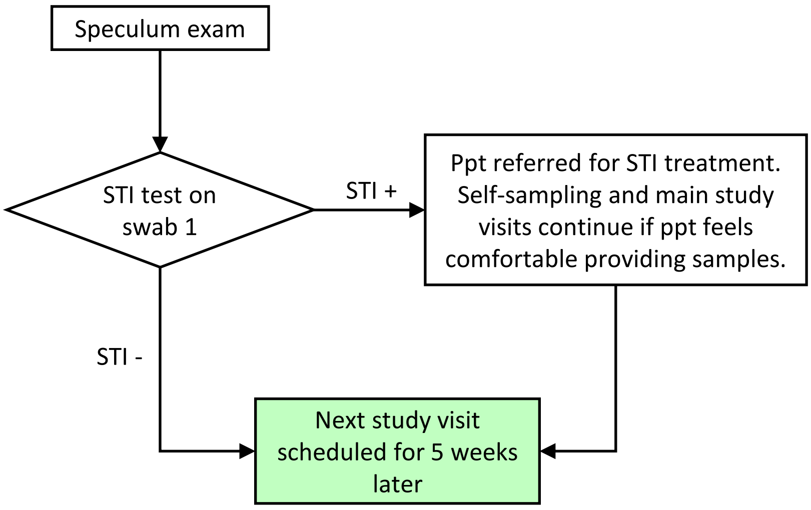

Samples collected before speculum exam:
Softcup (S)
Self-collected (SM) – lateral wall for microbiome
Samples collected during speculum exam:
Swab 1 – vulvo-vaginal for STI testing (collected before exam at KEMRI)
Swab 2 – high vaginal wall for storage
Swab 3 – lateral wall for BV, pH
Swab 4 – lateral wall for culture
Swab 5 – lateral wall for microbiome
Swab 6 – lateral wall for proteomics
Swab 7 – lateral wall for metabolomics
Swab 8 – endocervical cytobrush

Figure S1D

Exit (pre-exam):

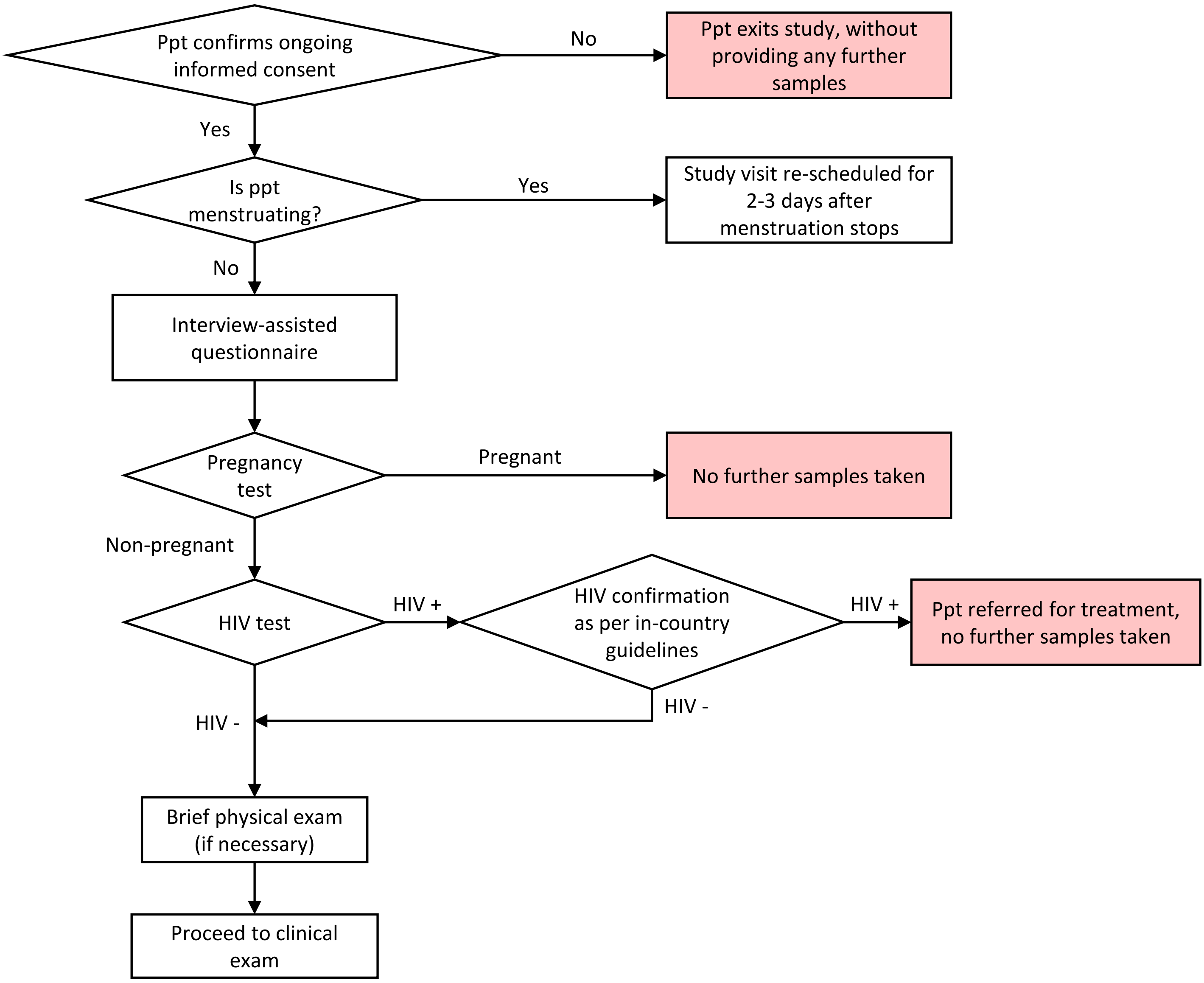

Clinical sampling procedures:

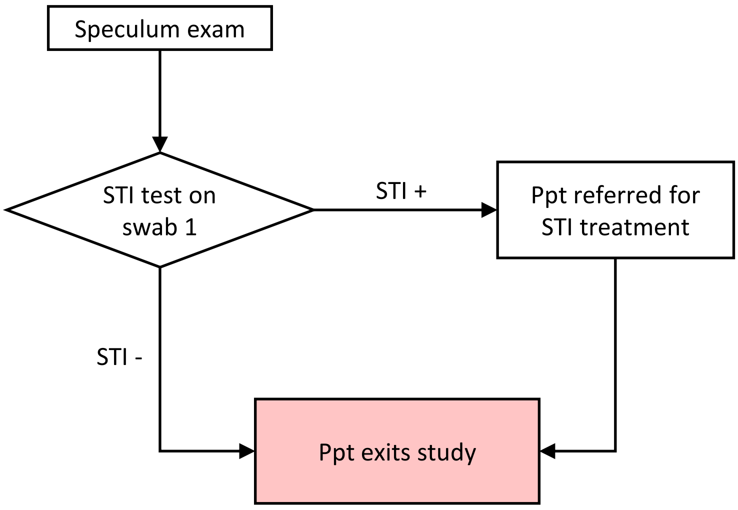

Samples collected before speculum exam:
Softcup (S)
Self-collected (SM) – lateral wall for microbiome
Samples collected during speculum exam:
Swab 1 – vulvo-vaginal for STI testing (collected before exam at KEMRI)
Swab 2 – high vaginal wall for storage
Swab 3 – lateral wall for BV, pH
Swab 4 – lateral wall for culture
Swab 5 – lateral wall for microbiome
Swab 6 – lateral wall for proteomics
Swab 7 – lateral wall for metabolomics
Swab 8 – endocervical cytobrush

Figure S2

The Menstrual Practices Questionnaire

For more information see www.menstrualpracticemeasures.org and the citation below.

Citation for the MPQ: Hennegan, J., Nansubuga, A., Akullo, A., Smith, C., & Schwab, K.J., (2020). The Menstrual Practices Questionnaire (MPQ): Development, elaboration, and implications for future research. *Global Health Action, 13*(1), 1829402. https://doi.org/10.1080/16549716.2020.1829402

Notes:

1. Question wording and terms should be adapted for different languages and contexts. In particular "menstrual materials" may need to be replaced with more familiar terminology or could be replaced using computerised filters to import the menstrual material/s used.
2. All questions refer to the last menstrual period. This can be facilitated through question wording (as shown below), or the time period could be specified at the start of questioning.
3. Not all questions are applicable for all respondents, appropriate filters should be applied. 4. Question order is presented here according to topic-groupings. The order should be modified as appropriate for delivery and may be integrated with other survey questions.

Question	Response options
**Menstrual material use**	
What type of underwear did you use during your last menstrual period?<br><br>*(select all that apply)*	• Bikini<br>• Boyshorts<br>• Thong<br>• Hipster<br>• G-string<br>• High-waisted<br>• Briefs<br>• Control top / shaper<br>• Tanga<br>• Brazilian<br>• French-cut<br>• Boxer briefs<br>• Period underwear<br>• Other (specify)
What type of material was your underwear made from during your last menstrual period?<br><br>*(select all that apply)*	• Cotton - natural fiber that is known for being soft, comfortable, and breathable.<br><br>• Polyester - synthetic fabric that is known for its durability and resistance to wrinkles.<br><br>• Nylon - synthetic fabric that is lightweight, strong, and has good elasticity. It is commonly used in clothing items such as stockings, swimwear, and activewear.<br><br>• Spandex - also known as Lycra or elastane, is a synthetic fabric that is known for its stretchability. It is commonly used in clothing items such as leggings, sports bras, and swimwear. Also known as control or shapewear.<br><br>• Lace - delicate fabric made from yarn or thread in an open web-like pattern. It is often used as a decorative element in clothing items such as lingerie, dresses, and tops.<br><br>• Satin - smooth, glossy fabric that is often made from

	silk, polyester, or nylon. It is commonly used in clothing items such as evening gowns, lingerie, and bedding.<br><br>• Silk - luxurious and soft natural fiber that is known for its luster and smooth texture.<br><br>• Microfiber - synthetic fabric that is known for being lightweight, soft, and moisture-wicking.<br><br>• Bamboo - is made from the pulp of bamboo plants and is known for being soft, breathable, and hypoallergenic.<br><br>• Period underwear - designed to be leak-proof and absorbent, making it an alternative to traditional pads or tampons during menstruation. It is typically made from a combination of materials such as cotton, polyester, and spandex.
What were all the materials you used to catch/absorb your menstruation when you were **at home** during your last menstrual period?<br><br>*(select all that apply)*	• Cloth/towel<br>• Disposable sanitary pad<br>• Reusable sanitary pad<br>• Toilet paper<br>• Cotton wool<br>• Mattress or foam<br>• Underwear alone<br>• Natural material (e.g., leaves, sand, grass)<br>• Period underwear<br>• Menstrual cup<br>• Tampon<br>• Other (specify)
What were all the materials you used to catch/absorb your menstruation when you were **away from home [at school/at work]** during your last menstrual period?<br><br>*(select all that apply)*	• Cloth/towel<br>• Disposable sanitary pad<br>• Reusable sanitary pad<br>• Toilet paper<br>• Cotton wool<br>• Mattress or foam<br>• Underwear alone<br>• Natural material (e.g., leaves, sand, grass)<br>• Period underwear<br>• Menstrual cup<br>• Tampon<br>• Other (specify)
[Cloth users] Were your cloths bought to be used for menstruation or used for something else first? *(select one)*	• Bought to be used during menstruation<br>• Used for something else first (e.g., clothes, bedding)<br>• Don't know
Did you wash and reuse any of your menstrual materials during your last menstrual period? *(select one)*	• No<br>• Yes

**Changing menstrual materials**

During your last menstrual period, how many times did you change your menstrual material on the heaviest day of your period? *(day = 24 hours) (select one)*	• 1 time (wear until the next day)<br>• 2 times (eg. morning and evening)<br>• 3 times (eg. morning, evening and once during day) • 4 times (eg. morning, evening, and twice during day) • More than 4 times
Where did you most often change your menstrual materials when you were **at home** during your menstrual last period?<br>*(select one)*	• Latrine<br>• Bedroom<br>• Bathroom/washing space (separate from latrine)<br>• Outside/bush/field<br>• Other (specify)
How often did you change your menstrual materials when you were **away from your home [at school/at work]** during your last menstrual period? (e.g., school, market, working outside the home)<br>(*select one*)	• Every day of my period<br>• Some days<br>• One day<br>• Never/no days
Where did you most often change your menstrual materials when you were **away from your home [at school/at work]** during your last menstrual period?<br>*(select one)*	• Latrine<br>• A bathroom (separate from latrine)<br>• Another room at the location<br>• Outside/bush/field<br>• Other (specify)
**Handwashing**	
Did you wash your hands **before** changing your menstrual materials during your last menstrual period?<br>*(select one)*	• Never<br>• Sometimes<br>• Every time
Did you wash your hands **after** changing your menstrual materials during your last menstrual period?<br>*(select one)*	• Never<br>• Sometimes<br>• Every time
**Genital washing**	
How often did you wash your genitals during your last menstrual period?<br>*(select one)*	• At the end of my period only<br>• Every 2-3 days<br>• Once per day<br>• Twice per day<br>• Three or more times per day
When you washed your genitals, did you use soap?<br>*(select one)*	• Never<br>• Sometimes<br>• Every time
**Disposal of menstrual materials**	
Where did you most often dispose of your used menstrual materials when you were **at home** during your last menstrual period?<br>*(select one)*	• Into the latrine/toilet<br>• Burned<br>• Household rubbish (bin in latrine)<br>• Household rubbish (bin not in latrine)<br>• Taken to community rubbish<br>• Buried/bush/waterway<br>• Did not dispose of any materials (including reusables)<br>• Other (specify)

Where did you most often dispose of your used menstrual materials when you were **away from your home [at school/at work]** during your last menstrual period? *(select one)*	• Transported home to dispose or reuse<br>• Into the latrine/toilet<br>• Bin in the latrine/toilet<br>• Bin onsite but outside of the latrine/toilet<br>• Community rubbish (not onsite)<br>• Bush/buried/waterway<br>• Burned<br>• Other (specify)
When disposing of your used menstrual materials, did you usually wrap them in anything? *(select one)*	• No<br>• Yes, plastic bag, cover of pad<br>• Yes, toilet paper<br>• Yes, cloth<br>• Yes, other
**Storage of menstrual materials**	
After your last menstrual period, did you keep [store] your menstrual materials? (includes leftover disposables or reusables) *(select one)*	• No<br>• Yes
Where did you store your menstrual materials after your last menstrual period? *(select one)*	• Cupboard, cabinet or drawer<br>• Under bed<br>• In the toilet/latrine room<br>• In a bathroom (not latrine/toilet)<br>• Other
Did you store your menstrual materials inside any wrapping, packaging or case? *(select one)*	• Wrapped in plastic or in plastic bag (including disposable pads in original packaging)<br>• Wrapped in fabric or in a fabric bag<br>• Wrapped in paper<br>• Cardboard box<br>• No wrapping or container<br>• Other
**Washing materials**	
Did you soak your materials when washing them during your last menstrual period? How long did you usually soak them for?	[Time]<br>0 is did not soak
Where did you most often wash your menstrual materials during your last menstrual period? *(select one)*	• Shared bucket (or similar container)<br>• Own bucket (or similar container)<br>• Shower/bath<br>• Sink/basin (inside home)<br>• Sink/basin (outside home)<br>• Well, spring, waterway<br>• Other
Did you use soap or detergent to wash or soak your menstrual materials during your last menstrual period? *(select one)*	• Never<br>• Sometimes<br>• Every time

Drying materials	
Where did you most often dry your menstrual materials during your last menstrual period? *(select one)*	• Outside (hanging)<br>• Outside (hidden)<br>• Inside (hanging)<br>• Inside (hidden)<br>• Other

When your menstrual materials were drying, did you usually cover them with anything? *(select one)*	• No<br>• Yes
When your menstrual materials were drying during your last menstrual period, were they in the sun? *(select one)*	• Never<br>• Sometimes<br>• All the time
During your last period, were your menstrual materials completely dry before you used them? *(select one)*	• Never<br>• Sometimes<br>• Every time
**Additional sterilisation practices**	
[Fabric reusables] Did you use an iron on your menstrual materials before you reused them during your last menstrual period? *(select one)*	• Never<br>• Sometimes<br>• Every time
[Menstrual cup users] Did you boil your menstrual cup during [or just before or after] your last period?	• No<br>• Yes
[Menstrual cup users] How many times did you boil your menstrual cup?	[number]
[Menstrual cup users] How long, on average, did you boil your menstrual cup?	[duration]
**Toilet/latrine practice during menstruation**	
During your last menstrual period, when you were **at home** did you use the same location for urination as when you do not have your menstrual period? *(select one)*	• Never<br>• Sometimes<br>• All the time
During your menstrual last period, when you were **at work** did you use the same location for urination as when you do not have your menstrual period? *(select one)*	• Never<br>• Sometimes<br>• Every time

This work is licensed under a Creative Commons Attribution-NonCommercial 4.0 International License

Figure S3A

<u>**VMRC4Africa - Pilot**</u>

An observational study to characterize vaginal microbial community interactions and obtain bacterial isolates from different geographies in Africa to contribute to the global effort to understand the functional microbial diversity that occurs in women with stable *L. crispatus*-dominant versus unstable vaginal microbiota.

Version 3.0
18 September 2023

INFORMED CONSENT FORM FOR SPECIMEN COLLECTION AND USE.

Note for Project Staff: If the person cannot read, this form must be read to her exactly as written, in her language of choice, in the presence of an impartial witness who must sign this form to confirm that the correct information was provided to the person and that the woman freely consents to have her specimens stored for possible future research.

PRINCIPAL INVESTIGATOR:
Associate Professor Jo-Ann Passmore
Mucosal Infection Group
Division of Medical Virology, Faculty of Health Sciences, University of Cape Town, Anzio Road, Observatory 7925, Cape Town, South Africa
Tel: +27 21 650 7963
Fax: +27 21 406 6681
Email: Jo-ann.Passmore@uct.ac.za
Website: https://passmore-lab.org.za/

INTRODUCTION:

We are doing a study to try to better understand what makes a vagina healthy. Women who have unhealthy vaginas can more easily get HIV (if they have sex with a person who has HIV) and have more trouble becoming pregnant, or have problems during pregnancy. We ask you to take part in this study because you live near the research site, are a woman, are sexually active, do not have HIV, are not pregnant and are between 18 and 40 years. Taking part in this study is completely voluntary (your choice) and you can decide not to take part without it affecting the care you may get at this clinic. You can also decide to stop the study at any point, but please let us know if this happens. We guess that about 100 women in South Africa will take part in this study, and another 100 in Kenya.

While you are part of the study, genital specimens will be collected and self-collected from you for sexual reproductive health testing and for research.

This document gives you information about the collection and use of your genital specimens. The project staff will talk to you about this information. If you have any questions feel free to ask the project staff. We will ask you to sign this document to show that you want to take part in the study. You will receive a copy of this document to keep or we can store this document for you here away from other project documents.

WHAT WILL HAPPEN IN THIS STUDY?

Screening visit

In the first study visit, you will have a pregnancy and HIV test. If your pregnancy and HIV tests are both negative, you will have a vaginal pH (acid level of the vagina) and bacterial vaginosis test (when the vagina's natural bacteria levels are out of balance), this is one vaginal swab (like a long cotton bud) you may collect yourself or the Nurse can do it for you, if you would like. If all four of the tests are negative, we will ask you some questions about your life and sexual behaviour. We will do a vaginal exam and collect samples using up to 5 swabs against the inside of your vagina. If you are pregnant,have HIV, or any signs of your vaginal bacteria being out of balance, you cannot take part in the study and we will not do a vaginal exam.

If you have the vaginal exam, we will use one swab to test if you have an STI, and another swab to re-test if you have bacterial vaginosis. If you have bacterial vaginosis, you cannot take part in the rest of the study. If you have an STI, we will offer treatment or a referral to your local clinic for treatment. After the treatment, we will do another test to see if your STI is gone. If it is gone, we need to wait for at least 28 days from the time of treatment and then you can take part in the study.

Longer study

If you take part in the longer study, it will involve three clinic visits over 10 weeks. We will try to have these visits after or before your work or other commitments. It is very important that we do not have these visits when you are having your period. You will do a pregnancy and HIV test. We will ask you to collect a vaginal swab yourself and answer some questions about your sexual behaviour. If you are not pregnant and don't have HIV, we will do a vaginal exam and collect samples using up to 8 cotton buds against the inside of your vagina. We will ask you to wear a menstrual cup inside your vagina for around 45 minutes. If you are pregnant or have HIV, we will not take any samples, but you can still take part in the interviews. We will test you for sexually transmitted infections.

If you have an infection, we will contact you and ask you to come back to the clinic for treatment. We may also send you to your local clinic for treatment. If you choose, we will also send your partner(s) for treatment, or you may bring them to the clinic so that we can treat them.

We will also ask you to take two weekly swabs yourself at home, 2-3 days apart, on the weeks you are not visiting the clinic (weeks 1-4 and 6-9). You can drop them off at the clinic when you have time. When you drop off your swabs at the clinic, we will ask you some quick questions about your week, which should take no more than 5-10 minutes. We will teach you how to take the swabs yourself. We will give you swabs to take home to do this. The swabs are stable at room temperature and do need to be kept in a cool dry place.

We will not tell you the results of your sample testing except for the results of sexually transmitted infections, HIV, bacterial vaginosis and pregnancy testing.

WHAT ARE THE RISKS?

The risks of this study are that you may feel uncomfortable during the vaginal exam and when the vaginal samples are taken. Our study team is well trained and will guide you on what to do. This makes the examination and sample collection as comfortable as possible. You may also feel uncomfortable when the study team ask you personal questions about your sexual behaviour. We do not judge you. You can choose to not answer any question that you feel uncomfortable with. But we hope that you will be willing to answer questions as truthfully as possible, so that we have accurate information about your health.

WHAT ARE THE BENEFITS?

You may benefit from knowing if you have a sexually transmitted infection or bacterial vaginosis. You will receive treatment at no cost. Treating infections in the vagina may reduce your risk of getting HIV or long-term complications, such as problems falling pregnant. You may also benefit from knowing your pregnancy and HIV status and being sent for appropriate care and treatment as needed..

It is unlikely that there will be any direct benefits to you from tests done on your stored specimens. There may be benefits to society and women in general from doing research on your genital specimens. These benefits may include learning more about the genital tract, new health conditions in the future and

development of products that promote women’s genital health.

YOUR SPECIMENS

Your genital specimens will be shipped and stored at special facilities that are designed to store specimens safely and securely for a long time. The storage facilities are based at the University of Cape Town. Only approved researchers can have access to the genital specimens in the storage facilities. Some staff of the storage facilities need to have access to your specimens to store them and to keep track of where they are, but these staff will not have information that directly identifies you.

Your genital specimens will be kept for the duration of study, in an Africa-specific biorepository. This is for research looking at helping women stay healthy, unless you separately agree to long term storage and future use.

HOW WILL GENITAL SPECIMENS BE USED?

Researchers from different centres will use the swabs. This includes the University of Cape Town, Desmond Tutu Health Foundation (DTHF), the Kenya Medical Research Institute (KEMRI) in Nairobi, and possibly elsewhere. The researchers may use your vaginal swabs to look for other infections, or what causes such infections, or at your body's response to infection, or for new research to develop products that improve women’s genital health. You need to agree to using your swabs for future research studies on a separate document.

You will not receive any monetary benefits or individual acknowledgement from the use of your samples in new research or to develop new products.

WHAT ABOUT CONFIDENTIALITY?

To keep your information private, your genital swabs will be labelled with a code.. Your personal information (name, address, phone number) will not be placed on the swabs. In the future, when researchers are given your stored swabs to study, they will only be given only the code. They will not be given your personal information.

The results of any tests done on your stored specimens will not be included in your health records. We will try as hard as possible to keep your personal information confidential, but we cannot guarantee absolute confidentiality. Your personal information may be disclosed if required by law.

WHAT ARE MY RIGHTS?

Agreeing to this research is completely voluntary (your choice). If you decide not to participate, this will not affect your care at the research centre or elsewhere. You can change your mind at any time. If you change your mind, you must contact your project doctor or nurse and let them know.

WHAT HAPPENS IF I GET HURT TAKING PART IN THIS STUDY?

This research study is covered by an insurance policy taken out by the University of Cape Town in the very unlikely event you suffer a bodily injury because you are taking part in the study.

The insurer will pay for all reasonable medical costs required to treat your bodily injury, according to the SA Good Clinical Practice Guidelines 2006, which are based on the Association of the British Pharmaceutical Industry Guidelines. The insurer will pay without you having to prove that the research was responsible for your bodily injury. You may ask the study doctor for a copy of these guidelines.

The insurer will *not* pay for harm if, during the study, you:

- Use medicines or other substances that are not allowed
- Do not follow the study nurse’s instructions
- Do not tell the study nurse that you have a bad side effect from the study procedures
- Do not take reasonable care of yourself

If you are harmed and the insurer pays for the necessary medical costs, usually you will be asked to accept that insurance payment as full settlement of the claim for medical costs. However, accepting this offer of insurance cover does not mean you give up your right to make a separate claim for other losses based on negligence, in a South African court.

It is important to follow the study nurse's instructions and to report straight away if you have a side effect from the study procedures.

WHAT DO I DO IF I HAVE QUESTIONS?

If at any time, you feel that you have any questions during the study, please do not hesitate to contact the study doctor or sister at _______________

The 24-hour telephone number that you can reach the doctor in charge or another authorized person is _______________

This study has been submitted to the University of Cape Town Human Research Ethics Committee (HREC) and the local country ethics review committee which have approved the study. The study has been planned following the guidelines in the Declaration of Helsinki, which tells doctors how to carry out medical research involving people. A copy may be obtained from the study clinicians should you wish to review it.

If you want any information regarding your rights as a research participant, or have complaints regarding this research study, you may contact: Dr Marc Blockman, University of Cape Town Human Research Ethics Committee Phone: 021 406 6346 or your local country ethics review committee as provided to you.

STATEMENT OF CONSENT:

Please read the statement below carefully and think about your choice. No matter what you decide, it will not affect your care.

I have been given information about this study and all my questions have been answered. I understand that this is voluntary. I agree to take part in this study.

- **YES**
- **NO**

SIGNATURES

____________________	____________________	____________
Participant's Name (print)	Participant's signature or thumbprint (if illiterate)	Date
____________________	____________________	____________
Study Staff Name (Print)	Staff signature	Date

WITNESS:

Signing below means that the participant whose thumbprint is above has had the informed consent form explained to her. It also means that I was present the whole time and that the details were being explained. I confirm that the participant had a chance to ask questions. The participant will be provided with a copy of this form to keep

____________________	____________________	____________
Witness Name (Print)	Witness signature	Date

The section below is to be completed by the person who administered the informed consent.

Was a copy of the signed consent form given to the participant:

- Yes
- No

If no, why not:

Figure S3B

VMRC4Africa - Pilot

An observation study to characterize vaginal microbial community interactions and obtain bacterial isolates from different geographies in Africa to contribute to the global effort to understand the functional microbial diversity that occurs in women with stable *L. crispatus*-dominant versus unstable vaginal microbiota.

Version 3.0
18 September 2023

INFORMED CONSENT FORM FOR SPECIMEN STORAGE, POSSIBLE FUTURE RESEARCH AND USE.

Note for Project Staff: If the person cannot read, this form must be read to her exactly as written, in her language of choice, and an impartial witness must sign this form to confirm that the correct information was provided to the person and that the woman freely consents to have her specimens stored for possible future research.

PRINCIPAL INVESTIGATOR:
Associate Professor Jo-Ann Passmore
Mucosal Infection Group
Division of Medical Virology, Faculty of Health Sciences, University of Cape Town, Anzio Road, Observatory 7925, Cape Town, South Africa

Tel: +27 21 650 7963
Fax: +27 21 406 6681
Email: Jo-ann.Passmore@uct.ac.za
Website: https://passmore-lab.org.za/

INTRODUCTION

This document gives you information about the storage, and use of your genital specimens and data for possible future research. The project staff will talk to you about this information. If you have any questions you should ask the project staff. We will ask you to sign this document to show whether or not you want your genital specimens and data kept for possible future research and/or product development. You will get a copy of this document to keep or if you prefer we can store this document for you here away from other project documents.

You can still take part in this project if you do not want the storage of your specimens and data.

WHAT WILL HAPPEN WITH MY SAMPLES AND DATA?

Before we do any further studies on your samples, we must get permission from a special committee. This committee will act on your behalf to make sure that your privacy is protected and your samples are only used for good purposes.

We may use some of the microbes that we find in your samples to make a new type of treatment (called a probiotic) which could one day be sold by a company. These may have value and may be developed and owned by the research team and/or others. If this happens, there are no plans to pay you if this happens.

We want to study the genetic material called RNA that is collected from a person's body. We hope by studying the RNA, we can learn more about how to protect young women from HIV. We will put information about the kind of genes your cells are making in a Africa specific biorepository, for genetic information. The Vaginal Microbiome Research Consortium for Africa (VMRC4Africa) controls this database. The biorepository will allow researchers to collect and share information with each other, which may result in learning new and important things more quickly. The information in this database will be stored forever.

Other researchers must get permission from a special committee to use your data in the biorepository. Qualified researchers who get permission to use information may not be from the University of Cape Town. Some may be from other institutions, universities or from commercial companies.

STORAGE OF GENITAL SPECIMENS AND DATA

Your genital specimens and data will be kept forever in the VMRC4Africa biorepository, for research looking at helping women stay healthy.

YOUR PRIVACY

We will label all your information with a study code number. We will store the list that connects your name to your study code in a locked cabinet or in a secure computer file. We will do our very best to protect your information.

Your privacy is very important to us. If you decide to allow us to store your data, we will use many safety measures to keep your genetic information safe. However, we cannot guarantee that you will never be identified. We think it is very unlikely that this happens but it is possible that someone could

- Break into the computer system(s). They could then find the code that links your genetic and medical information together. This is very unlikely.
- Find a way to link your genetic or medical information in a database back to another member of your family. Your genetic information is unique to you. But you share some genetic information with your children, parents, brothers, sisters and other blood relatives. So it might be possible for someone to use genetic information from your relatives to help figure out who you are. Again, it is unlikely this would happen.

OTHER POSSIBLE HARMS

Genetic information could also be used by law enforcement agencies to identify a person or his/her blood relatives. There could be other privacy risks we do not know about.

WHAT ARE MY RIGHTS?

Agreeing to your genital specimens and data to be stored for future research is your choice. You can decide not to have any specimens or data stored for future research. Even if you decide now that your specimens and data can be stored for possible future research and/or product development, you can change your mind at any time. If you change your mind, no more information about you will be collected. However, we will not be able to take back the information that has already been looked at in the research. If you change your mind, you must contact your study doctor or nurse and let them know.

BENEFITS

There are no benefits to you to take part in this study. We will not share any individual genetic results with you. There may be benefits to society and women in general of doing research on your genital specimens. These benefits may include learning more about the genital tract, new health conditions in the future and development of products that promote women's genital health.

WHAT DO I DO IF I HAVE QUESTIONS?

If at any time, you feel that you have any questions during the study, please do not hesitate to contact the study doctor or sister at _______________

The 24-hour telephone number that you can reach the doctor in charge or another authorized person is _______________

This study has been submitted to the University of Cape Town Human Research Ethics Committee (HREC) and the HREC has approved of this study. The study has been planned following the guidelines in the Declaration of Helsinki, which tells doctors how to carry out medical research involving people. A copy may be obtained from me should you wish to review it.

If you want any information regarding your rights as a research participant, or have complaints regarding this research study, you may contact: Dr Marc Blockman, University of Cape Town Human Research Ethics Committee Phone: 021 406 6346

PERMISSION AND SIGNATURE - VMRC4AFRICA BIOREPOSITORY:
We told you above about the other uses for your genital specimens and data and how information will be shared outside of this study. Please **choose only 1 option below.** Whatever you choose, we will keep track of your decision and how your samples may be used.

- **I allow my extra samples and information to be used for other studies related to vaccines, the immune system, coronavirus, HIV and other diseases. This may include genetic testing and keeping my cells growing over time.**

- **I agree to the option above and ALSO to allow my extra samples and information to be used in studies that look at the whole genome.**

- **I do NOT allow my extra samples to be used in any other studies. This includes NOT allowing genetic testing, growing more of my cells or studies that look at my whole genome.**

If you agree to your specimen storage and future use you will need to sign below. Before you sign, make sure of the following:

- You have read the consent form, or someone has read it to you.
- You feel that you understand what the study is about and what will happen to your samples and data if you join. You understand what the possible risks and benefits are.
- You have had your questions answered and know that you can ask more.
- You agree to join this study.

You will not be giving up any of your rights by signing this form.

SIGNATURES

______________________	______________________	_____________
Participant's Name (print)	Participant's signature or thumbprint (if illiterate)	Date

______________________	______________________	_____________
Study Staff Name (Print)	Staff signature	Date

WITNESS:

Signing below means that the participant whose thumbprint is above has had the specimen storage for future research process explained to her. It also means that I was present the whole time and that the details were being explained. I confirm that the participant had a chance to ask questions. The participant will be provided with a copy of this form to keep

Witness Name (Print)	Witness signature	Date

The section below is to be completed by the person who administered the informed consent.

Was a copy of the signed consent form given to the participant:

- Yes
- No

If no, why not:
