# Supplementary material for: Vaginal Microbiome Research Consortium for Africa: study protocol of a multicentre prospective clinical study to evaluate temporal vaginal microbial composition associated with maintenance of reproductive health in women in South Africa and Kenya
Source: BMJ Open. 2025 Feb 22;15(2):e090938. doi: 10.1136/bmjopen-2024-090938 (PMC11848679; doi:10.1136/bmjopen-2024-090938)
Supplement: Supplementary file 2 [file bmjopen-2024-090938.reviewer_comments.pdf]

PEER REVIEW HISTORY

BMJ Open publishes all reviews undertaken for accepted manuscripts. Reviewers are asked to complete a checklist review form and are provided with free text boxes to elaborate on their assessment. These free text comments are reproduced below.

ARTICLE DETAILS

Title (Provisional)

Vaginal microbiome research consortium for Africa: Study protocol of a multi-centre prospective clinical study to evaluate temporal vaginal microbial composition associated with maintenance of reproductive health in women in South Africa and Kenya

Authors

Kullin, Brian Ronald; Gitome, Serah; Happel, Anna-Ursula; Pidwell, Tanya; Lefevre, Mellissa; Madikida, Anda; Wekesa, Pauline; Mahlangu, Karabo; Ochieng, James; Awili, Lydia; Agolla, Winnie; Otieno, Rhoda; Mutharimi, Amos; Ganief, Yacoeb; Daniels, Rezeen; Chicken, Anika; Welp, Kirsten; Livingstone, Hannah; Swanepoel, Caleb; Claassen-Weitz, Shantelle; Kanyoka, Pride; Ravel, Jacques; Humphrys, Michael; Bilski, Lisa; Mulder, Nicola; Bekker, Linda Gail; Gill, Katherine; Jaspan, Heather; Bukusi, Elizabeth; Passmore, Jo-Ann

---

VERSION 1 - REVIEW

**Reviewer**	**1**
**Name**	**Brusselaers, Nele**
**Affiliation**	**Karolinska Institutet, Molecular medicine and Surgery**
**Date**	**03-Sep-2024**
**COI**	**None**

Impressive project, and I do look forward to the scientific papers coming out as it will be a very valuable resource. In the discussion part you state it will inform African scientists, but I think it will inform scientists across the global. I am really pleased to see we will soon have large-scaled information on the vaginal microbiome from other settings than Europe and the US.

I do have a few suggestions for clarifications as it is a complex project with many steps, and somethings were not always clear. Anyway, congratulations with the project as it seems you should be close to finalising enrolment

- I think it would be good to add the total (max) number of samples that will be collected, as it is not mentioned in the abstract – and the presented numbers were not always what I

expected. On page 37 you see max 19 self-collected swabs (19 * 200 = 3,800), while on page 16 you state you will have 5000 self-collected swabs; and if it would be twice-weekly throughout it would be 20*200=4000… It is likely you won't have full sample collection for everyone, but it would be nice to see the estimates consistently

- In the abstract you write bi-weekly which implies every 2 weeks, while you are collecting samples every 2 weeks! Twice a week? Ok, apparently bi-weekly can be used for both "twice a week" as "every two weeks" (https://www.grammarly.com/blog/biweekly/) but it is confusing, so better to be clear?

- I expected HPV to be mentioned earlier on in the intro or methods description. It seems an important factor in women's health – and you do plan to collect this information, so stress importance more? Will you collect information on HPV vaccination or is it not yet (widely) available in both settings? Are there cervical cancer screening programs in place already? Do you know anything about prior HPV carriage or cervical screening results?

- In objectives/hypothesis I would already specify how you defined BV, as different definitions have been used.

- I was wondering about the inclusion criteria about sexual activity – what is your rationale? Comparibility?

- Table 2: please explain [SM] and [S]. You do refer to Table 3, but it does not make me much wiser. Note_ Table 2 was not entirely visible as probably in horizontal format, while the pdf only included vertical format…

- Regarding the menstrual cups: you will ask all women to collect vaginal fluids during the nurse visits –(at enrolment – week 5 – week 10). Please explain this procedure in a bit more detail. How long does it have to be in place to collect a sufficient amount of fluid? I do see on the figure on page 37 that you will collect 11 samples during the nurse visits – or no, it is confusing as the questionnaires are also presented with a dot (can't you use another symbol for questionnaires as the colour is very similar to the vulvo-vaginal swab?). Can't you use same colour coding and symbols in Table 2?

- Timing of sample collection: from Table 2 it seems you start with the speculum exam, then ask for a self-collected sample (when she is on the chair??) and then the nurse can continue the other sample? And somewhere the menstrual cup comes in… Just trying to understand the logistics… And it is a nurse who decides if the cervix looks OK and there are no signs of STI… Are nurses sufficiently trained to make this distinction/diagnosis? Or is it first a clinician, than self-sampling, then nurse?

- I hope you can collect some minimal information on the women who drop out on the way because they are not eligible (anymore)?

- I really like the paragraph on ethical considerations. Important!

**Reviewer**	**2**
**Name**	**Lee, Heung Kyu**
**Affiliation**	**Korea Advanced Institute of Science and Technology (KAIST), Dept. of Biological Sciences**
**Date**	**06-Dec-2024**
**COI**	**I have no competing interests.**

The protocol presented for the VMRC4Africa study is well-crafted, clearly structured, and presents a highly relevant research initiative with significant potential for advancing our understanding of the vaginal microbiome in sub-Saharan Africa. The study's design, involving parallel cohorts from both South Africa and Kenya, offers a robust and geographically diverse approach to evaluating the dynamics of vaginal microbial communities. The choice of 16S rRNA gene sequencing for bacterial communities and ITS sequencing for fungi ensures a comprehensive characterization of microbiota across time, which is essential for identifying key factors related to vaginal health and inflammation.

The study's goal to establish a biorepository of Lactobacillus isolates from these regions is a commendable strategy with clear implications for the development of targeted, region-specific biotherapeutic products to address conditions like bacterial vaginosis. The ethical approval and commitment to data transparency through public repositories further enhance the study's rigor and scientific value.

Overall, the VMRC4Africa study represents a timely and innovative effort to address an important gap in the understanding of women's health in sub-Saharan Africa, and the protocol is written in a clear, organized, and methodologically sound manner. This is an excellent contribution to the field of microbiome research and holds great promise for improving health outcomes in the region.

VERSION 1 - AUTHOR RESPONSE

Reviewer: 1
Dr. Nele Brusselaers, Karolinska Institutet

Comments to the Author:
Impressive project, and I do look forward to the scientific papers coming out as it will be a very valuable resource. In the discussion part you state it will inform African scientists, but I think it will inform scientists across the global. I am really pleased to see we will soon have large-scaled information on the vaginal microbiome from other settings than Europe and the US.
I do have a few suggestions for clarifications as it is a complex project with many steps, and somethings were not always clear. Anyway, congratulations with the project as it seems you

should be close to finalising enrolment

- I think it would be good to add the total (max) number of samples that will be collected, as it is not mentioned in the abstract – and the presented numbers were not always what I expected. On page 37 you see max 19 self-collected swabs (19 * 200 = 3,800), while on page 16 you state you will have 5000 self-collected swabs; and if it would be twice-weekly throughout it would be 20*200=4000… It is likely you won't have full sample collection for everyone, but it would be nice to see the estimates consistently

We acknowledge that the way the swab numbers are calculated could be clarified and we have updated the manuscript to explicitly state these numbers. We have also updated the 'Sample size' section to include estimates of the numbers of women who will be screened and provide initial samples (n = 600). With these in mind, the total swab numbers are calculated based on:

DNA - 1 swab taken at screening (600 x 1)

2 swabs taken at the 3 main visits (200 x 3 x 2)

2 swabs taken per week for 8 weeks (200 x 8 x 2)

Cervicovaginal - 2 swabs taken at screening (600 x 2)

6 swabs taken at 3 main visits (200 x 3 x 6)

Menstrual cup - 1 taken at screening (600 x 1)

1 taken at the 3 main visits (200 x 3 x 1)

- In the abstract you write bi-weekly which implies every 2 weeks, while you are collecting samples every 2 weeks! Twice a week? Ok, apparently bi-weekly can be used for both "twice a week" as "every two weeks" (https://www.grammarly.com/blog/biweekly/) but it is confusing, so better to be clear?

We have updated the abstract to reflect the fact that the self-collected swabs are collected twice a week.

- I expected HPV to be mentioned earlier on in the intro or methods description. It seems an important factor in women's health – and you do plan to collect this information, so stress importance more? Will you collect information on HPV vaccination or is it not yet (widely) available in both settings? Are there cervical cancer screening programs in place already? Do you know anything about prior HPV carriage or cervical screening results?

We have included an additional paragraph on HPV in the introduction to emphasise its importance. We are, unfortunately, not collecting prior vaccination data in this study, but despite vaccination programmes having been established in both South Africa and Kenya (2014 and 2019, respectively), vaccine uptake rates are fairly low in both populations (10-40%). We do not perform cervical cancer screening during the clinical exam and would not be able to definitively diagnose CIN. However, any cervical abnormalities (other than those associated with STIs) that require further evaluation are deemed ineligible and these women are referred to the public health facilities for evaluation and further management.

- In objectives/hypothesis I would already specify how you defined BV, as different definitions have been used.

We have defined BV (Nugent 0-3) in the updated 'Objectives and Hypothesis' section

- I was wondering about the inclusion criteria about sexual activity – what is your rationale? Comparibility?

This is correct. We chose to only enrol sexually active women due to the significant effect that sexual activity has on the vaginal microbiome. These women make up the majority of the participant population and are the most likely to benefit from an intervention with a live biotherapeutic product. *Lactobacillus* spp. that demonstrate stable persistence in women, despite sexual activity are likely better candidates for such a product.

- Table 2: please explain [SM] and [S]. You do refer to Table 3, but it does not make me much wiser. Note_ Table 2 was not entirely visible as probably in horizontal format, while the pdf only included vertical format…

The 'SM' and 'S' samples have been explained in more detail in footnotes to Table 3 in the revised manuscript.

- Regarding the menstrual cups: you will ask all women to collect vaginal fluids during the nurse visits –(at enrolment – week 5 – week 10). Please explain this procedure in a bit more detail. How long does it have to be in place to collect a sufficient amount of fluid? I do see on the figure on page 37 that you will collect 11 samples during the nurse visits – or no, it is confusing as the questionnaires are also presented with a dot (can't you use another symbol for questionnaires as the colour is very similar to the vulvo-vaginal swab?). Can't you use same colour coding and symbols in Table 2?

We thank the reviewer for the useful suggestions and have updated the text to provide more details on the menstrual cup collections. We have also updated Figure 1 to include a different symbol for the questionnaires and Table 2 to have similar symbol shapes and colours to Figure 1.

- Timing of sample collection: from Table 2 it seems you start with the speculum exam, then ask for a self-collected sample (when she is on the chair??) and then the nurse can continue the other sample? And somewhere the menstrual cup comes in… Just trying to understand the logistics… And it is a nurse who decides if the cervix looks OK and there are no signs of STI… Are nurses sufficiently trained to make this distinction/diagnosis? Or is it first a clinician, than self-sampling, then nurse?

We have updated the ordering of events in Table 2 and the main visit description to make the procedure clearer. At both sites, the study nurses are sufficiently trained to conduct pelvic exams and identify abnormalities. Abnormalities are referred to the clinical officer and/or study clinician for further treatment and antibiotic prescription.

- I hope you can collect some minimal information on the women who drop out on the way because they are not eligible (anymore)?

We collect all study information up until their last visit. We also capture their reason for exiting the study prior to completion. For those women who become ineligible for sampling (due to pregnancy or HIV acquisition) we allow them to continue in the study should they choose to. These women can continue to attend the visits and complete the study questionnaires. We have updated the 'Data Handling' paragraph to include capturing reasons for early exit.

- I really like the paragraph on ethical considerations. Important!

Reviewer: 2
Dr. Heung Kyu Lee, Korea Advanced Institute of Science and Technology (KAIST)
Comments to the Author:
The protocol presented for the VMRC4Africa study is well-crafted, clearly structured, and presents a highly relevant research initiative with significant potential for advancing our understanding of the vaginal microbiome in sub-Saharan Africa. The study's design, involving parallel cohorts from both South Africa and Kenya, offers a robust and geographically diverse approach to evaluating the dynamics of vaginal microbial communities. The choice of 16S rRNA gene sequencing for bacterial communities and ITS sequencing for fungi ensures a comprehensive characterization of microbiota across time, which is essential for identifying key factors related to vaginal health and inflammation.
The study’s goal to establish a biorepository of Lactobacillus isolates from these regions is a commendable strategy with clear implications for the development of targeted, region-specific biotherapeutic products to address conditions like bacterial vaginosis. The ethical approval and commitment to data transparency through public repositories further enhance the study’s rigor and scientific value.
Overall, the VMRC4Africa study represents a timely and innovative effort to address an important gap in the understanding of women's health in sub-Saharan Africa, and the protocol is written in a clear, organized, and methodologically sound manner. This is an excellent contribution to the field of microbiome research and holds great promise for improving health outcomes in the region.
